# Uveal Melanoma-Derived Extracellular Vesicles Display Transforming Potential and Carry Protein Cargo Involved in Metastatic Niche Preparation

**DOI:** 10.3390/cancers12102923

**Published:** 2020-10-11

**Authors:** Thupten Tsering, Alexander Laskaris, Mohamed Abdouh, Prisca Bustamante, Sabrina Parent, Eva Jin, Sarah Tadhg Ferrier, Goffredo Arena, Julia V. Burnier

**Affiliations:** 1 Cancer Research Program, Research Institute of the McGill University Health Centre, 1001 Decarie Blvd, Montreal, QC H4A 3J1, Canada; thupten.tsering@mail.mcgill.ca (T.T.); Alexander.laskaris@mail.mcgill.ca (A.L.); mohamed.abdouh@muhc.mcgill.ca (M.A.); prisca.bustamantealvarez@mail.mcgill.ca (P.B.); sabparent@icloud.com (S.P.); eva.jin@mail.mcgill.ca (E.J.); sarah.ferrier@mail.mcgill.ca (S.T.F.); goffredo.arena@mcgill.ca (G.A.); 2 Ospedale Giuseppe Giglio Fondazione San Raffaele Cefalu Sicily, 90015 Cefalu, Italy; 3 Mediterranean Institute of Oncology, 95029 Viagrande, Italy; 4 Experimental Pathology Unit, Department of Pathology, McGill University, QC H3A 2B4, Canada

**Keywords:** Uveal melanoma, extracellular vesicles, liver metastasis, liquid biopsy, mass spectrometry

## Abstract

**Simple Summary:**

Uveal melanoma is a rare but deadly cancer that shows remarkable metastatic tropism to the liver. Extracellular vesicles (EVs) are nanometer-sized, lipid bilayer-membraned particles that are released from cells. In our study we used EVs derived from primary normal choroidal melanocytes and matched primary and metastatic uveal melanoma cell lines from a patient. Analysis of these EVs revealed important protein signatures that may be involved in tumorigenesis and metastatic dissemination. We have established a model to study EV functions and phenotypes which can be used in EV-based liquid biopsy.

**Abstract:**

Extracellular vesicles (EVs) carry molecules derived from donor cells and are able to alter the properties of recipient cells. They are important players during the genesis and progression of tumors. Uveal melanoma (UM) is the most common primary intraocular tumor in adults and is associated with a high rate of metastasis, primarily to the liver. However, the mechanisms underlying this process are poorly understood. In the present study, we analyzed the oncogenic potential of UM-derived EVs and their protein signature. We isolated and characterized EVs from five UM cell lines and from normal choroidal melanocytes (NCMs). BRCA1-deficient fibroblasts (Fibro-BKO) were exposed to the EVs and analyzed for their growth in vitro and their reprograming potential in vivo following inoculation into NOD-SCID mice. Mass spectrometry of proteins from UM-EVs and NCM-EVs was performed to determine a protein signature that could elucidate potential key players in UM progression. In-depth analyses showed the presence of exosomal markers, and proteins involved in cell-cell and focal adhesion, endocytosis, and PI3K-Akt signaling pathway. Notably, we observed high expression levels of HSP90, HSP70 and integrin V in UM-EVs. Our data bring new evidence on the involvement of UM-EVs in cancer progression and metastasis.

## 1. Introduction

Uveal Melanoma (UM) is the most common primary intraocular tumor in adults [1,2], and the second most common type of melanoma. It develops within the uveal tract of the eye, most frequently in the choroid [3,4]. Although there has been tremendous progress in understanding the genetic landscape [5,6,7,8], diagnosis [9,10,11], and treatment [12,13] of UM, the overall survival rate has not changed in the last three decades [14]. Its annual incidence is estimated at 3.75 and 5.2 cases per million individuals in Canada and the United States, respectively [15,16], while in Europe it varies according to latitude (2–8 cases per million) [17]. While the rate of UM occurrence is relatively low, approximately 50% of patients develop metastasis, primarily to the liver (90%) [18]. The 1-year survival rate of UM patients dramatically drops to 15% once it metastasizes [13,19] due to the absence of effective treatments and the high tumor burden at the time of detection [20]. While metastases are rarely detected at UM primary diagnosis, evidence has shown that circulating tumor cells can be found at diagnosis, suggesting that systemic involvement is an early phenomenon [21]. Moreover, the mechanisms underlying this process are not well understood. This implies that current clinical surveillance tools are not sensitive enough to detect premetastatic stages thereby underscoring the need for better and more sensitive biomarkers to complement and validate existing clinical surveillance. Extracellular vesicles (EVs) have been shown to harbor selective biomarkers in various cancers and to provide valuable clinical information [22]. However, the role of EVs as biomarkers and mediators of metastasis has not been widely explored in UM.

EVs are nanoparticles emitted under physiological and pathological conditions such as cancer. They are highly heterogeneous based on their size, shape and subcellular origin [23]. Minimal information for the study of EVs have now been standardized [24]; these include and are not limited to the size, floating density, as well as the presence of classical markers such as tetraspanins, annexins, Alix, and heat shock proteins (HSPs) [25,26]. The underlying molecular mechanism involved in EV formation, delivery of cargo inside EVs and ultimately in their release is still not clear [26,27]. In contrast, their uptake by target cells has been shown to occur via numerous pathways (i.e., endocytosis, micropinocytosis, phagocytosis) [28,29]. EVs are loaded with RNA [30,31], DNA [27,32], lipid [33,34] and proteins [35,36], and play a vital role in intercellular communications [23,24,37,38]. Notably, cancer-derived EVs promote cell proliferation, migration, invasion, angiogenesis and metastases [39,40,41].

A growing body of evidence proposes that EV cargo could be used as circulating biomarkers in liquid biopsy-based platform, particularly in the context of cancer. miRNA profiling of UM-derived exosomes has been performed [42,43]. In addition, the proteome profile of UM secretome and that of UM-derived EVs have also been reported [22,44,45]. However, none has addressed protein differential expression between healthy- and UM-derived EVs.

We performed this study to investigate the effects of EVs derived from UM cell lines on the behaviour of target cells, and to compare the protein contents in EVs derived from UM cells and normal choroidal melanocytes (NCMs). We have previously demonstrated that blood-derived EVs from patients with ocular and cutaneous melanoma are uptaken by and reprogram single oncosuppressor-mutated (SOM) cells into malignant cells [46,47,48]. Here, we showed that as opposed to NCM-EVs, UM-EVs increased the proliferation of target SOM cells such as BRCA1-deficient fibroblasts (Fibro-BKO) and induced their malignant transformation. In addition, proteomic analyses showed that UM-derived EVs were enriched in proteins involved in cell-cell and focal adhesion, endocytosis, and metastatic niche organization. Altogether, these data shed light on the role of EVs in driving cancer progression, and their potential use in a liquid biopsy platform to monitor patients affected by UM.

## 2. Results

### 2.1. Primary NCMs Were Efficiently Cultured from Human Eyes

UM can arise de novo or from pre-existing benign nevi, stemming from malignant transformation of melanocytes of the uveal tract, mainly the choroid. As NCMs are not available commercially, and in order to perform comparative analyses of UM-EVs and to study their effects, we established primary NCM cultures as a control counterpart. NCM cultures were established from 3 donors as described under Material and Methods section (Figure S1 and Table S1). Geneticin was used to suppress growth of retinal pigmented epithelium (RPE) cells and fibroblasts, thus ensuring pure NCM cultures, as previously described [49]. The majority of NCM cells displayed a spindle morphology with brown pigmented cytoplasmic granules (Figures S1 and S2A). In addition, these cells stained positive for MART-1/Melan A, vimentin, S100 and HMB45, which is an indicator of melanogenesis [49] (Figure S2B,C). This suggests that our purification protocol yielded pure NCMs. Of note, EVs derived from donors 2G.PPccF1968Y and 2G.PPXG1981Y (NCM cells) were used for NanoSight, transmission electron microscopy (TEM), and Western blots. EVs derived from donors 2G.PPwC1963Y and 2G.PPXG1981Y (NCM cells) were used for mice experiments (Table S1B).

### 2.2. EVs Derived from UM Cells and NCMs are Efficiently Internalized by Target Cells

**Figure 1:**
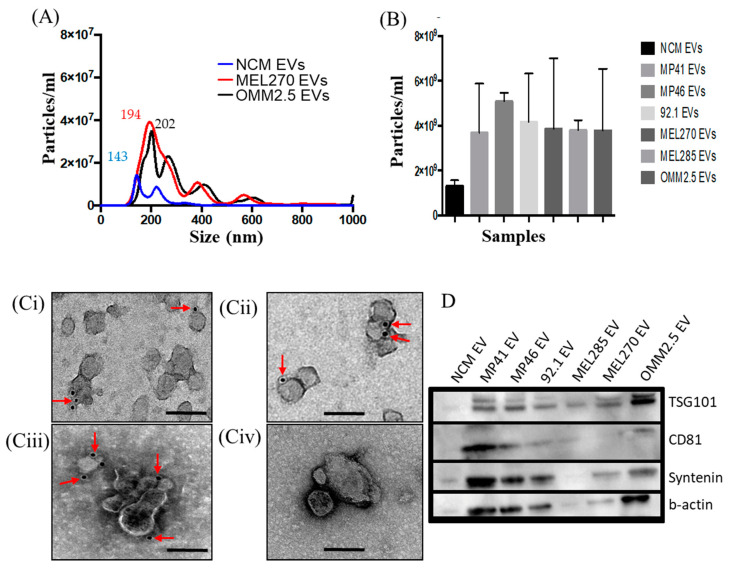
Characterization of extracellular vesicles (EVs) derived from normal choroidal melanocytes (NCMs) and Uveal Melanoma (UM) cells. (**A**,**B**) Nanoparticle tracing analysis NTA of EVs derived MEL270, metastatic UM cells (OMM2.5) and NCMs. (**B**) NTA data showing concentrations of EVs from different cell sources. Data are expressed as mean ± SD (*n* = 3). (**C**) Representative micrographs of immunoGold-TEM on MP46-EVs (**Ci**–**Cii**) 92.1-EVs (**Ciii**) and NCM-EVs (**Civ**) labelled with a cocktail of antibodies against CD81 (**Ci**), TSG101 (**Cii**) and CD63 (**Ciii**) (red arrows). Scale bars 200 nm. (**D**) Proteins isolated from EVs derived from different cell sources were analyzed by Western blot for the expression of specific exosome markers.

As our first goal was to determine the effects of EVs on target cells, we characterized the nature of these particles, and determined their behaviour when added to target cell cultures. EVs were isolated both from NCMs and from established UM cell cultures. Selected UM cell lines show typical UM-initiating mutations (characteristics shown in Table S1B) and we used MEL270 and OMM2.5, which are matched primary and metastatic, respectively, cell lines derived from the same UM patient. We confirmed the identity of the EVs both physically and phenotypically. As assessed by NanoSight for nanoparticle tracing analysis (NTA) and TEM, the isolated EVs were round-shaped vesicles with a mean diameter of 181 nm for UM-EVs (mean range 163 nm (MEL270) to 214 nm (MP46)), and 278 nm for EVs from NCMs (Figure 1A). As expected NCMs emitted a lower amount of EVs compared to the UM cell lines. (Figure 1B). In addition, by using TEM-based immunogold labeling and Western blot, we observed that these vesicles expressed selective markers of exosomes (i.e., CD81, CD63, TSG101) (Figure 1C,D). Detailed analyses of NTA and TEM of EVs are shown in Figures S3A–J and S4A–J respectively.

**Figure 2:**
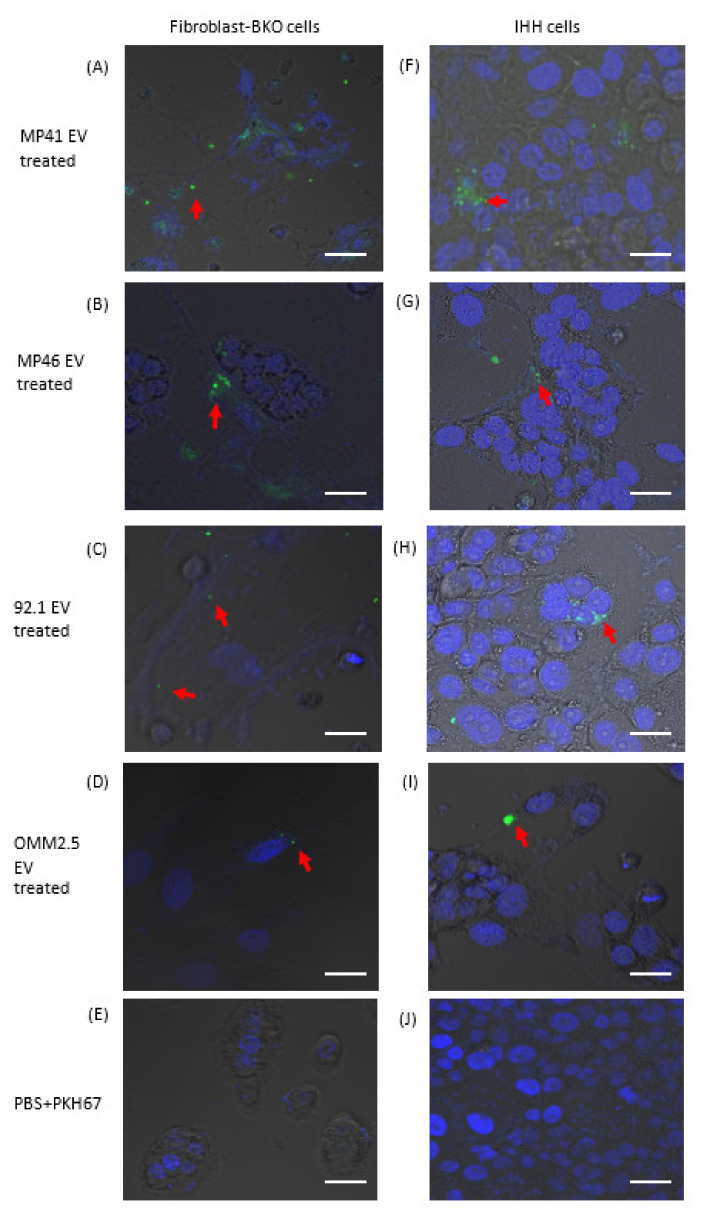
Isolated EVs were efficiently internalized by target cells. EVs from UM cells and NCMs were labeled with PKH67 dye and purified using Optiprep dentity gradient and ultracentrifugation. PKH67-labeled EVs were added to Fibro-BKO cells (**A**–**D**) and IHH (**F**–**I**). EV uptake (red arrows) was monitored under confocal microscopy 6 h later. (**E**,**J**) Cells were exposed to PKH67 solution processed as was the case for labeled EVs. Scale bars 20 μm.

In order to exert their effects, EVs must be internalized by target cells and deliver their cargo. To assess EV uptake by target cells, we tagged them with PKH67. To eliminate excess free PKH67 which may interfere with interpretation of EV uptake, labeled EV preparations were passed through an Optiprep density gradient (Figure S5A). NTA, immunogold TEM, and Western blot analyses revealed enrichment of PKH67-labeled EVs in fraction 3 that was devoid of free-floating dye (Figure S5B–E). Following incubation of purified PKH67-tagged EVs with Fibro-BKO and immortalized human hepatocytes (i.e., IHH; used herein because the liver is the primary site for UM metastasis [19,50]), we observed that treated cells efficiently internalized EVs (Figure 2A–D and 2F–I, respectively). In contrast, cells incubated with preparations from PKH67 samples without EVs did not show any green puncta signal in either Fibro-BKO (Figure 2E) or IHHs (Figure 2J). These data show that pure pools of EVs from UM cells are efficiently internalized by target cells.

### 2.3. Exposure to UM-EVs Increases the Proliferation, Migration and Invasion of Fibro-BKO Cells

**Figure 3:**
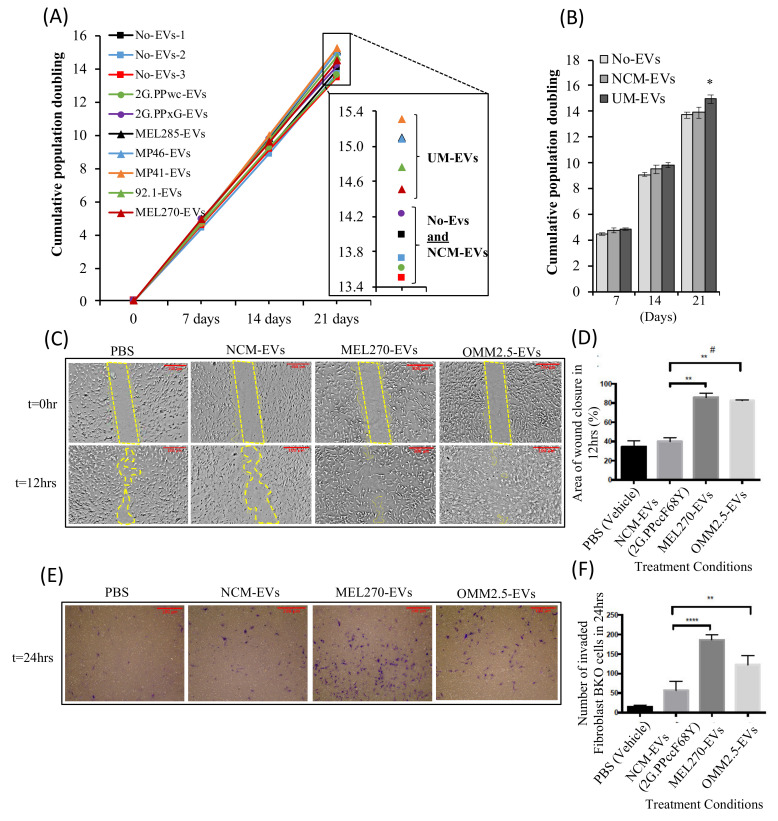
UM-EVs promote proliferation, migration and invasion of BRCA1-deficient fibroblast (Fibro-BKO) cells. (**A**–**C**) Fibro-BKO cells were cultured for 3 weeks in the presence of culture medium without EVs (No-EVs), NCM-EVs (from PPWc and PPxG eye donors) or UM-EVs. (**A**,**B**) Cells were analyzed for their growth potential by measuring population doubling capability at every passage. Data in inserts represent cumulative population doublings at the end of the treatment periods (**A**). Column graphs represent pooled data from three EV-free medium (No-EVs) samples, two NCM-EVs preparations and five UM-EVs preparations. Data are mean ± SD. *p* values ˂ 0.05 (*) (**B**). (**C**–**F**) Fibro-BKO cells were cultured for 12 h (**C**,**D**; cell migration) or 24 h (**E**,**F**; cell invasion) in the presence of culture medium without EVs (PBS), NCM-EVs or UM-EVs. Data are mean  ±  SD. (**D**) *p* value = 0.0086 (**), *p* value = 0.0051 (#). (**F**) *p* value = 0.0072 (**), *p* value < 0.0001 (****), MEL270 UM-EVs/OMM2.5 UM-EVs, *p* value = 0.0033 (**) (*n* = 3). Scale bar:100 µm, magnification 100×.

EVs regulate different biological functions via the transfer of their cargo into target cells [46,47,51,52,53,54]. We have previously demonstrated that blood-derived EVs from patients with ocular and cutaneous melanoma are uptaken by and reprogram single oncosuppressor-mutated (SOM), such as Fibro-BKO cells into malignant cells [46,47,48]. We wanted to investigate the behavior of Fibro-BKO cells and analyze their growth potential when exposed to either culture medium without EVs (No-EVs), NCM-EVs or UM-EVs. Fibro-BKO cells were treated for 3 weeks, and their population doubling levels (i.e., PDL) were measured at successive cell passages. NCM-EVs did not affect the behavior of Fibro-BKO cells when compared to cells treated with EV-free culture medium (Figure 3A,B). When compared to Fibro-BKO cells exposed to NCM-EVs, cells treated with UM-EVs displayed increased proliferation as shown by the increase in cell PD, an effect that was significant at 3 weeks of exposure (1 PD increase with a range of 0.5 to 1.39) (Figure 3A,B). In parallel, we verified if exposure to cancer EVs altered cell viability in vitro. During the length of cell exposure (i.e., 3 weeks), we did not observe any effect of cancer cell-derived EVs on the percentage of viable cells (Figure S6).

Furthermore, we sought to investigate the effect of EVs on Fibro-BKO migration and invasion. EVs derived from NCM (2G.PPccF68Y) and UM cells (MEL270 and OMM2.5) were incubated with Fibro-BKO for 12 h and 24 h to analyze cell migration and invasion, respectively (Figure 3C–F). Fibro-BKO cells treated with UM-EVs exhibited higher migration and invasion compared to Fibro-BKO cells treated with NCM-EVs or only Phosphate Buffer Saline (PBS, vehicle). The migratory and invasion capacity of UM-EVs treated Fibro-BKO cells were approximately two times higher than those treated with NCM-EVs or PBS (Figure 3C–F). Moreover, Transwell invasion assay suggested that primary uveal melanoma derived EVs (MEL270) displayed enhanced invasion capability compared to metastatic uveal melanoma EVs (OMM2.5) (Figure 3E–F). Overall, these results indicate that UM-EVs enhance the proliferation, migration and invasion capabilities of Fibro-BKO cells compared to control groups (NCM-EVs or PBS).

### 2.4. Fibro-BKO Cells Treated with UM-EVs Promote Tumor Growth In Vivo

**Figure 4:**
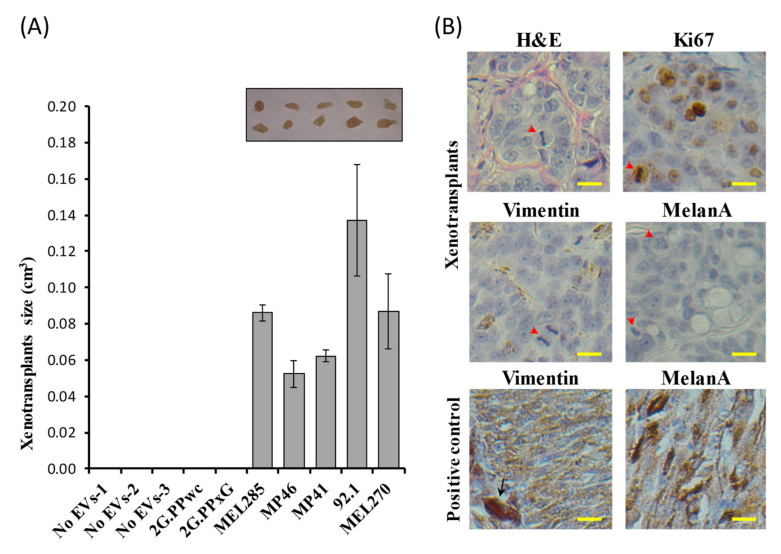
In vivo tumorigenicity assay of Fibro-BKO cells treated with UM-EVs (**A**)Exposed cells were injected subcutaneously into NOD/SCID mice that were monitored for 4 weeks for tumors growth. At euthanasia, developing tumors were excised and their sizes were measured. (**B**) Formalin-fixed paraffin-embedded tumors were processed for H&E staining, and immunolabeled with anti-Ki67, anti-Vimentin and anti-MelanA antibodies. Scale bar: 10 µm. Red arrowheads pointed to mitotic figures, and black arrow pointed to a melanophage. Positive controls are from choroidal melanoma specimens.

Cancer EVs have been reported to regulate cancer invasion and metastasis [52,55,56,57,58,59]. Furthermore, we have previously shown that cancer EVs carrying mutated DNA and RNA induced malignant transformation of Fibro-BKO [60]. We analyzed the transforming abilities of UM-EVs on Fibro-BKO cells: at the end of a 3-week exposure to culture medium without EVs (No-EVs), NCM-EVs or UM-EVs, Fibro-BKO cells were inoculated subcutaneously into NOD/SCID mice. Mice injected with Fibro-BKO treated with EV-free culture medium or NCM-EVs did not develop any visible tumors at euthanasia (4 weeks following inoculation). In contrast, all mice injected with Fibro-BKO cells exposed to UM-EVs developed tumors with varying sizes (Figure 4A). Histopathological analyses of developing xenotransplants displayed features of adenocarcinomas (hematoxylin and eosin (H&E) staining) showing mitotic figures and high proliferation index (80–90% Ki67 positivity) (Figure 4B). Notably, we observed that UM-EVs-treated cells had completely changed their fate since developing tumors stained negative for vimentin, which is normally expressed on fibroblasts (Figure 4B). However, independent of UM-EVs used, we did not observe any positivity for MelanA, suggesting that fibroblast are refractory of phenotypic switch. Together, these data show that cancer UM-EVs significantly enhanced target Fibro-BKO cells proliferation, migration and invasion in vitro and induced their malignant transformation when injected into mice.

### 2.5. UM-EVs Carry Proteins Involved in Metastatic Niche Formation

**Figure 5:**
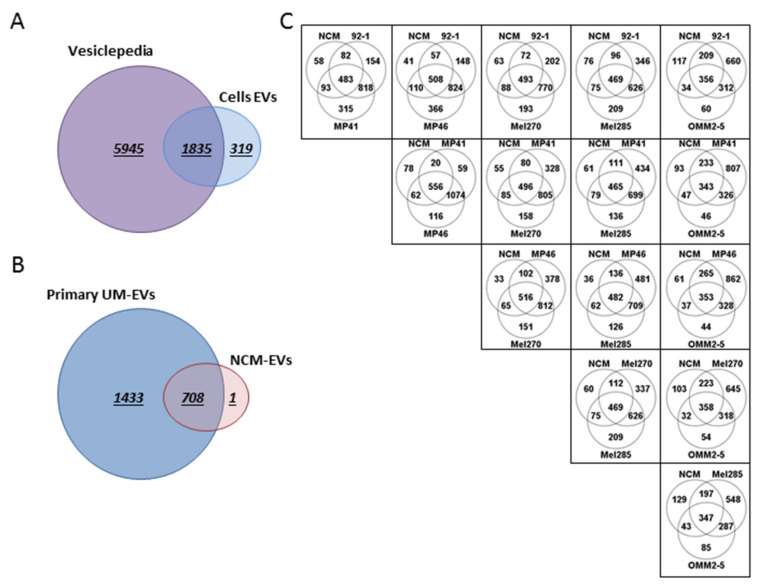
UM-EVs and NCM-EVs carried different sets of proteins. Venn diagram analyses. (**A**) The majority of proteins isolated from EVs derived from UM cells and NCMs were shared with data published in Vesiclepedia database. (**B**) NCM-EVs and primary UM-EVs shared 708 proteins, while 1 and 1433 proteins were exclusively present in NCM-EVs and primary UM-EVs, respectively. (**C**) Based on their protein cargo, primary UM-EVs clustered differently from NCM-EVs. A total of 20 to 265 proteins were exclusively shared between NCM-EVs and primary UM-EVs, while 287 to 1074 proteins were exclusively shared between EVs isolated from the different primary UM lines.

To gain an in-depth understanding of EV protein cargo isolated from both UM cells and NCMs, we performed a whole proteome analysis by quantitative mass spectrometry (MS). We undertook these analyses to determine putative factors that might explain the effects we observed following treatments of target cells with EVs, in particular the factors that might underlie UM metastasis. Using a quantitative proteomic analysis, we identified 2154 proteins of which 1835 (85%) overlap with EV proteins previously reported in the Vesiclepedia database [61]. In addition, we reported 319 novel proteins now added to the Vesiclepedia database (Figure 5A). Of the identified proteins, 33.06% (708 proteins) were shared between NCM-EVs and UM-EVs, whereas 66.93% (1433 proteins) were exclusive to UM-EVs and 1 protein was exclusive to NCM-EVs (Figure 5B). The shared proteins between the two datasets included typical EV protein signatures such as ESCRT components TSG101, CD81, CD63, CD9, and syntenin. Notably, each UM-EV set shared more proteins with other UM-EVs than with NCM-EVs. Comparing amongst cell lines, 60–80% of EV proteins from each UM cell line were found to overlap with every other UM cell line. In contrast, an average 33% of EV proteins from any UM cell line overlapped with NCM-EV proteins, which allows the conclusion that UM-EV cargo clustered differently from NCM-EV cargo (Figure 5C). In addition, tyrosinase related protein 1 (TYRP1; which is critical in the melanin biosynthesis pathway), melanotransferrin (MELTF) and melanocyte protein (PMEL) were present in almost all EV samples. Moreover, vimentin (VIM; an intermediate filament protein that is overexpressed in epithelial tumors such as UMs), melanoma-associated antigens D1 and D2 (MAGED1 and MAGED2) and melanoma antigen (MLANA) were present mainly in UM-EVs when compared with NCM-EVs. Altogether, these data indicate that the isolated EVs have a melanocytic origin.

**Figure 6:**
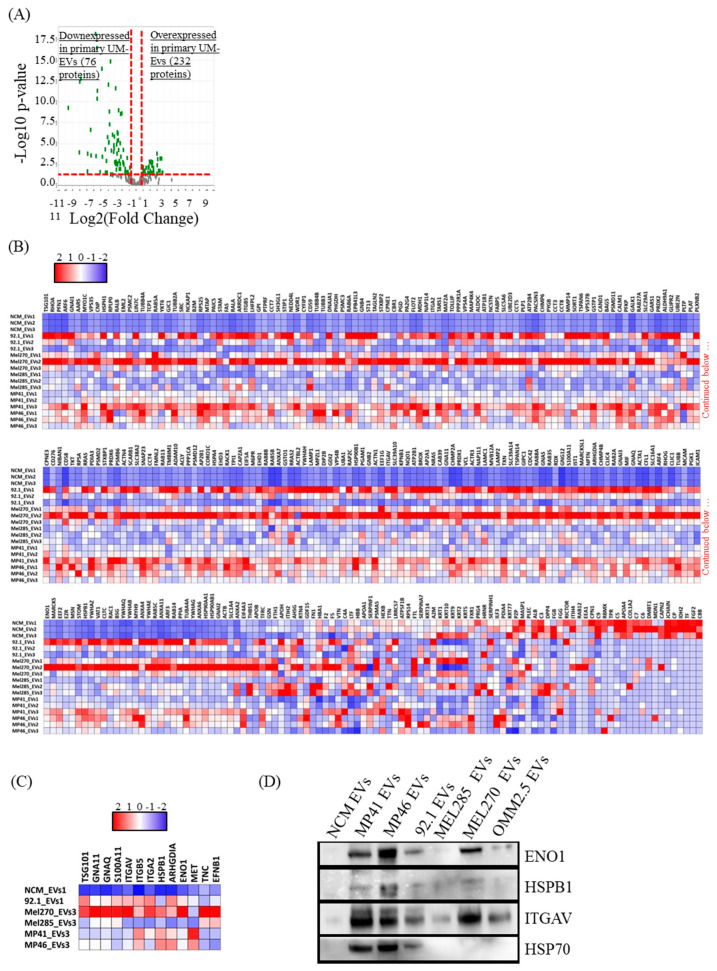
Primary UM-EVs are enriched in proteins involved in the regulation of tumor growth, homeostasis and metastasis organotropism. (**A**) Volcano plot representation of 308 proteins significantly and differentially expressed between primary UM-EVs and NCM-EVs. (**B**) Heatmap chart representing the 308 differentially expressed proteins. Note that primary UM-EV contents clustered differently from that of NCM-EVs. The full list of proteins is shown in Table S2. (**C**) Heatmap chart depicting the relative expression levels of proteins linked to tumorigenesis, cancer homeostasis, and metastasis organotropism. (**D**) Immunoblot validation of the relative expression levels of some proteins that emerged from MS data mining (see **C**). Note: HSP90 shown (Figure S7 (**E**)) was probed on a different membrane. In **B**,**C**, the key color represents Log(2) of protein quantitative ration where blue and red refer to downexpressed and overexpressed proteins in UM-EVs, respectively.

**Figure 7:**
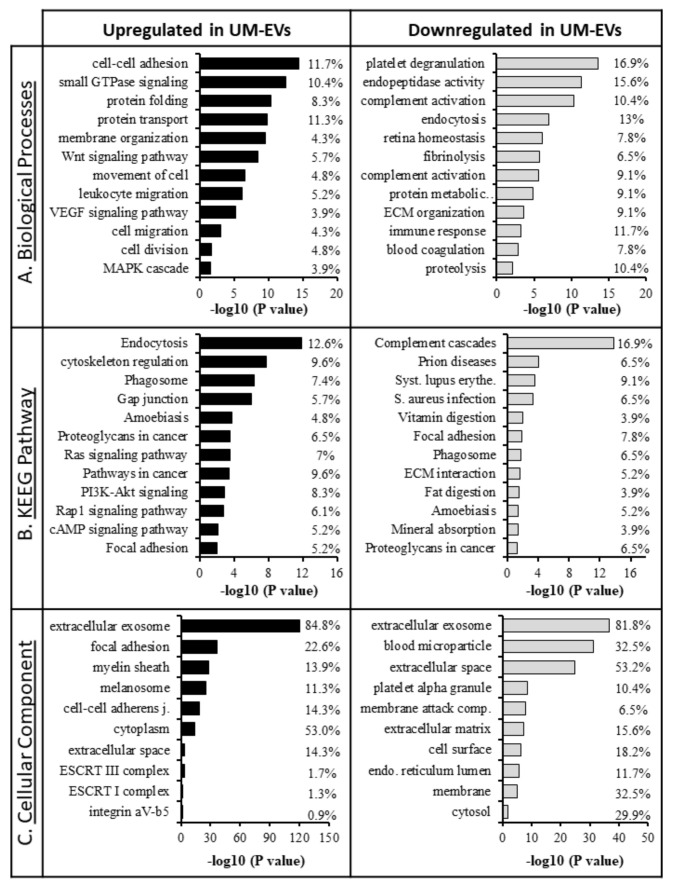
Gene ontology GO classification of proteomic data for differentially expressed proteins in primary UM-EVs and NCM-EVs. The most enriched categories in (**A**) biological process, (**B**) molecular function, and (**C**) cellular component. Left panel—UM-EVs, right panels—NCM-EVs.

Extensive comparisons of EV cargo in primary UM cell lines and NCMs were conducted (Figure 6). In relation to NCM-EVs, we found that 232 proteins were upregulated and 76 proteins were downregulated in UM-EVs (Figure 6A,B). Of the upregulated proteins, 200 were exclusively present in UM-EVs (Table S2). To identify the physiological processes to which these EVs-derived proteins are implicated, we clustered the most differentially expressed proteins (i.e., 232 overexpressed and 76 down-expressed in UM-EVs) into gene ontology (GO) categories using the DAVID bioinformatics platform (Table S4). Characterization by biological process highlighted categories consistent with the known functions of EVs. Upregulated proteins primarily clustered in the categories of cell-cell adhesion, small GTPase mediated signal transduction, and movement of cell or subcellular component (Figure 7A). Various other categories were present with lower protein counts but similar significance values (*p* < 0.05). These included leukocyte transendothelial migration, signaling cascades (VEGFR, Wnt, MAPK), cell division and migration. In contrast, down-regulated proteins clustered mainly in homeostatic processes such as endocytosis, immune response, retina homeostasis and platelet degranulation (Figure 7A). Molecular functions clustering using Kyoto Encyclopedia of Genes and Genomes (KEGG) pathway analysis revealed that UM-EVs were enriched in proteins involved in cell motility and cellular transit (actin cytoskeleton), cellular uptake (endocytosis, phagocytosis), and cancer associated signaling pathways (PI3K-Akt, Ras, Rap1, cAMP, Ras) (Figure 7B), whereas proteins related to immune escape of cancer, such as those involved in complement and coagulation cascades, were downregulated (Figure 7B).

Appropriately, when clustering the proteins based on cellular component, we found that the majority clustered into extracellular exosomes (i.e., EVs), for both upregulated and down-regulated groups (Figure 7C). There were certain cellular components exclusively present in the upregulated group, including ESCRT complexes I and III, and the integrin alpha v beta v (ITGαvβv) complex that were found to be significant (*p* < 0.05). UniProt tissue expression analysis [62] associated the UM-EVs proteins with Cajal-Retzius cells, B-cell lymphoma, brain, epithelium, liver and lung. In the down-regulated EV protein group, no proteins were found to be associated with the lungs and 35 proteins were clustered with the liver category, while the upregulated proteins had 58 total proteins in liver and 62 in lung categories.

We then mined our data by focusing on proteins that regulate tumor growth and those that modulate the metastatic niche environment (Figure 6C). The majority of upregulated proteins have been previously linked to tumorigenesis or cancer homeostasis, including signaling molecules (integrin αV, GNAQ, GNA11, the latter two being associated with UM tumorigenesis), molecular chaperon (HSPB1), and an ESCRT-I complex subunit (i.e., TSG101). Interestingly, integrin αV was associated with liver metastasis organotropism [56]. In addition, alpha-enolase (ENO1) was 11 times more expressed in UM-EVs than in NCM-EVs. This protein is a cancer cell surface biomarker found in EVs from melanoma and non-small cell lung carcinoma cells, and is expected to be used as a biomarker for many tumors [63,64]. Further, the chemoattractant S100A11 and ARHGDIA (RhoGDP) were upregulated in UM-EVs. Their expression is commonly increased in tumors and are often associated with tumor progression [65,66]. We validated the proteomic data by analyzing the expression of key proteins using immunoblotting (i.e., ENO1, HSPs, HSPB1 and integrin αV). Western blot analyses confirmed the pattern of protein expression as displayed in the heatmap chart from proteomic analyses (Figure 6D).

### 2.6. Metastatic UM-EVs Display Different Protein Expression Patterns Compared to Primary UM-EVs

**Figure 8:**
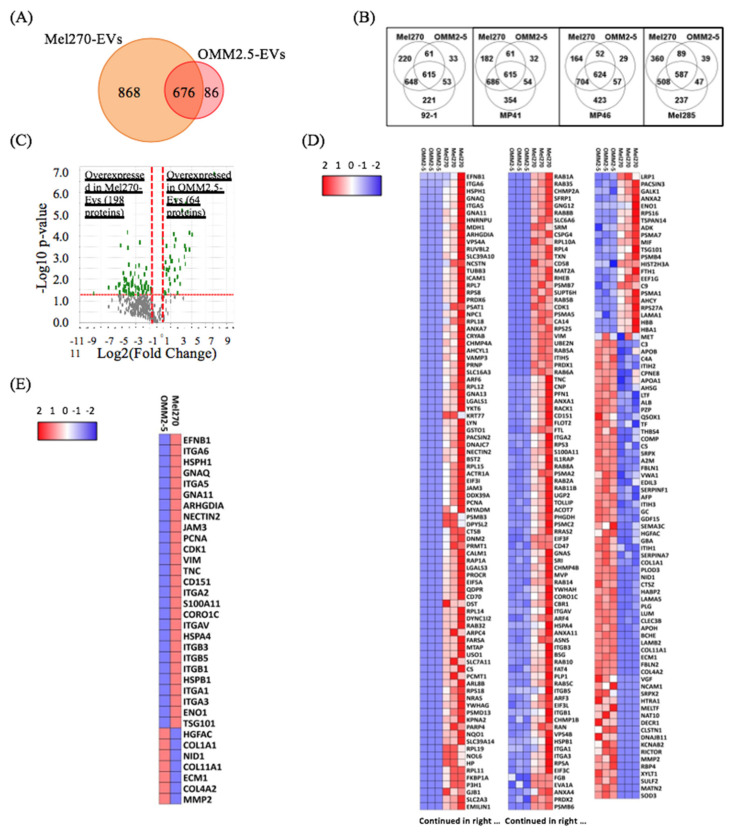
Primary UM-EV protein cargo clustered differently from that of metastatic UM-EVs. (**A**,**B**) Venn diagram analyses. Primary and metastatic UM-EVs shared 676 proteins, while 868 and 86 proteins were exclusively present in primary MEL270 UM-EVs and metastatic OMM2.5 UM-EVs, respectively (**A**). Based on their protein cargo, primary UM-EVs clustered differently from metastatic UM-EVs. 47 to 89 proteins were exclusively shared between primary UM-EVs and metastatic UM-EVs, while 508 to 704 proteins were exclusively shared between EVs isolated from the different primary UM lines (**B**). (**C**) Volcano plot representation of 262 proteins significantly and differentially expressed between primary and metastatic UM-EVs (MEL270 vs. OMM2.5). (**D**) Heatmap chart representing the 262 differentially expressed proteins. Note that primary MEL270 UM-EV contents clustered differently from that of metastatic OMM2.5 UM-EVs. The full list of proteins is shown in Table S3. (**E**) Heatmap chart depicting the relative expression levels of proteins linked to metastasis organotropism and metastasis regulation. In (**D**,**E)**, the key color represents Log(2) of protein quantitative ration where blue and red refer to downexpressed and overexpressed proteins, respectively.

To further analyze the differential protein cargo between primary and metastatic UM-EVs, we compared the expression of proteins in MEL270 primary UM cells and its metastatic derivative OMM2.5. Our analyses identified 1630 proteins of which 676 (42%) were shared, while 868 (53%) and 86 (5%) were exclusively expressed in MEL270 UM-EVs and OMM2.5 UM-EVs, respectively (Figure 8A). Notably, all primary UM-EVs shared more proteins with MEL270 UM-EVs (508 to 704 proteins) than with OMM2.5 UM-EVs (47 to 89 proteins), suggesting that primary UM-EV cargo clustered differently from metastatic UM-EV cargo (Figure 8B), and that these cargo regulated different cellular processes depending on donor UM cells (primary vs. metastatic).

When compared to OMM2.5-derived EVs, we found that 198 proteins were upregulated and 64 proteins were downregulated in MEL270-derived EVs (Figure 8C,D). Of the upregulated proteins, 116 were exclusively present in MEL270 UM-EVs (Table S3).

To determine the physiological processes associated with both primary and metastatic UM-EVs proteins, DAVID bioinformatics platform was utilized once more to functionally categorize proteins that demonstrated significant differential expression patterns between our primary UM cell line (MEL270) and the metastatic counterpart (OMM 2.5) (i.e., 198 overexpressed and 64 down-expressed in MEL270 UM-EVs secretions) (Table S5A,B). When referring to biological processes, the proteins upregulated in MEL270 UM-EVs clustered into leukocyte migration, small GTPase mediated signal transduction, integrin and tumor necrosis factor mediated signaling pathways, and MAPK cascade.

**Figure 9:**
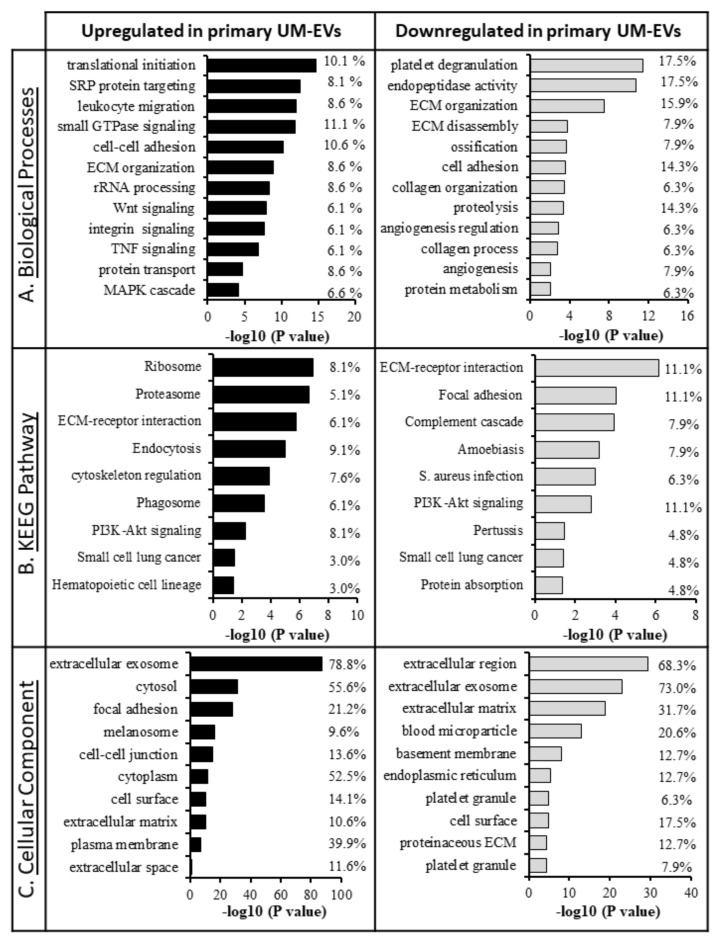
GO classification of proteomic data for differentially expressed proteins in primary MEL270 UM-EVs and metastatic OMM2.5 UM-EVs. The most enriched categories in (**A**) biological process, (**B**) molecular function, and (**C**) cellular component. Left panel—primary UM-EVs, right panels—metastatic UM-EVs.

In contrast, the down-regulated proteins took part in platelet degranulation, extracellular matrix (ECM) organization and disassembly, ossification, and cell adhesion (Figure 9A). KEGG pathway analysis revealed upregulated proteins were mainly clustered into cellular uptake and processing (proteasome, endocytosis, phagocytosis), ECM-receptor interactions, and hematopoietic cell lineage, whereas downregulated proteins clustered mainly into ECM-receptor interaction, collagen and coagulation cascades, and immune responses. PI3K-Akt signaling and proteins commonly associated with small cell lung cancer were seen in both up and down-regulated protein groups (Figure 9B). Clustering proteins by cellular components found an enrichment for extracellular exosome associated proteins, consistent with previous data (Figure 9C).

When we mined the differentially expressed proteins by focusing solely on those involved in metastasis regulation (Figure 8E), we found that overexpressed proteins in MEL270 UM-EVs belong to factors involved in metastasis organotropism (different classes of integrins, Coronin 1C and CD151), and mainly liver metastasis (i.e., ITGA5/B5) [56]. Other overexpressed proteins in MEL270 UM-EVs are involved in growth regulation (i.e., PCNA and CDK1) [67,68]. In contrast, we found that overexpressed proteins in OMM2.5 UM-EVs are those implicated in extracellular matrix (ECM) organization at metastatic niche sites. These include collagens, ECM1 and matrix metalloproteases (i.e., MMP2) [69,70,71]. These data suggest that while proteins carried in primary UM-derived EVs mainly helped in metastatic organotropism, those transported by metastatic counterparts were more involved in the maintenance of the metastatic niche.

## 3. Discussion

UM is the only malignancy in which diagnosis is made by clinical examination and generally without a biopsy. Unfortunately, the very limited number of metastatic UM cases that are deemed appropriate for surgical resection limits the possibility to obtain surgical samples that can be analyzed to understand the metastatic process. Given the high mortality rate, the asymptomatic nature and the lack of monitoring biomarkers, a liquid biopsy platform would be extremely valuable in the diagnosis and treatment of UM. Attempts have been made to profile miRNA contents of UM-isolated exosomes [42,43], as well as the proteome from both UM secretome and UM-derived EVs [22,44,45]. However, research focusing on reliable and clinically valuable metastatic biomarkers in UM has remained limited, and an in-depth analysis of the protein composition of EV cargo in UM is virtually not available. Furthermore, studies addressing the differential expression pattern of EV cargo proteins between normal melanocytes and melanoma cells have been neither done nor published. It was already known that circulating EV levels increase with advancing stages of cancer, suggesting potential roles in cancer progression and invasion [72]. Furthermore, we previously reported that EVs from different malignancies carry oncogenic factors that trigger malignant transformation in target cells [60]. In the present study, we wanted to verify whether EVs isolated from UM cell lines would trigger malignant transformation of Fibro BKO. Moreover, we wanted to perform a label-free LC-MS/MS analyses on proteins isolated from EVs derived from both UM cells and NCMs to determine potential factors that may underlay the observed biological effects and to apply the findings as a base for a liquid biopsy platform.

Considering that UM arises from melanocytes of the uveal tract [13] and due to the lack of a commercial source of normal uveal melanocytes we established a control line for comparative analyses isolating NCMs from the choroid tissue of donor eyes. The identity of NCMs was confirmed at both structural (cell shape) and phenotypical levels using a set of specific markers [49,73].

In our experiments we demonstrated that not only were UM-EVs efficiently internalized by Fibro BKO cells, but we also confirmed that these cells undergo dramatic changes after exposure to EVs as shown by increased proliferation, migration, invasion and acquisition of malignant characteristics. Moreover, factors carried by EVs belong to different molecular categories (i.e., DNA, mRNA, miRNA, proteins) and their roles in cancer biology have been extensively highlighted [32,74,75,76,77]. Recently, we provided evidence that cancer EVs actively transfer mutated cancer genes to target cells as well as a bulk of coding and non-coding RNAs acting as modulators of essential cellular pathways that impact cancer growth and progression [60]. Herein, we decided to deepen these analyses by focusing on UM-EV protein cargo.

The proteomic analyses performed in this study confirmed the differential expression of several proteins involved in cancer cell growth, movement and adhesion, and metastatic niche remodeling. Although relatively rare, UM is a deadly disease mainly as a result of the high risk of metastases occurring primarily in the liver [18,78]. Our analysis revealed several typical proteins implicated in the establishment of premetastatic niche that were differently expressed in the EVs from UM cells compared to those from NCMs. It has previously been reported that tumor-derived EVs expressing ITGαvβv are implicated in liver metastasis organotropism [56]. We observed high integrin αV protein levels in all UM-EVs analyzed when compared to NCM-EVs. Concurrently, DAVID bioinformatic analysis demonstrated that key proteins involved in the ITGαvβv complex were statistically significant in our dataset (*p* < 0.05). In relation to the integrins present in our UM-EV samples, there was upregulation of various signal transduction molecules such as S100A. It has been demonstrated that when integrins carried in cancer EVs were internalized by target cells, they activate SRC phosphorylation and pro-inflammatory S100 gene expression [56]. Further, EVs from melanoma were found to upregulate S100 proteins in recipient target organ cells resulting in vascular leakiness and promotion of metastasis [79]. Taken together, this suggests that UM-EVs promote a tumor induced inflammatory response and metastatic niche formation. Such data may provide insight into UM’s remarkable tropism to the liver.

Additionally, the expression patterns of both HSP90 and ENO1 were found uniformly increased across all UM-EVs. HSP90 is a molecular chaperone reported to be of crucial importance in cancer cell growth and survival owing to its involvement in promoting the MAPK and P13K/AKT pathways [80,81]. In similar fashion, ENO1 is linked to the AKT signaling pathway, is involved in promoting gastric cancer cell proliferation and metastasis and serves as a potential biomarker for certain cancers [72,74]. The PI3K-AKT signaling pathway was also highlighted in our KEGG pathway analysis as our UM-EVs contain a number of proteins linked to this signaling cascade. Other proteins involved in the process of metastasis were also identified in a set of UM-EVs (i.e., hepatocyte growth factor receptor tyrosine kinase (MET, in MP41 UM-EVs and MP46 UM-EVs), tenascin C (TNC, in MEL270 UM-EVs and MEL285 UM-EVs), ephrin-B2 (EFNB2, in MEL285 UM-EVs)) [82,83,84]. However, their expression was not uniformly increased in EVs derived from all UM cells.

Notably, when we mined for proteins differentially expressed between primary and metastatic UM-EVs (MEL270 UM-EVs vs. OMM2.5 UM-EVs), we found that primary UM-derived EVs were enriched for proteins involved in the regulation of cell growth (i.e., PCNA and CDK1) [67,68] and in metastatic organotropism (i.e., integrins, Coronin 1C and CD151) [56,85,86]. Coronin 1C is highly expressed in invasive human cancers and correlates positively with increased metastatic risk [85]. CD151 is a tetraspanin associated with tumor metastasis, and is correlated with poor prognosis, decreased overall survival and increased recurrence [86]. Similarly, HSPB1 [87] was also increased in EVs derived from MEL270. HSPB1 has been reported to play an important role in UM micrometastasis [87] and acts as switch between tumor dormancy and tumor progression in breast cancer [88]. In contrast, proteins transported by metastatic UM-EVs are involved in the maintenance of the metastatic niche, mainly ECM modeling and organization (i.e., collagens, ECM1 and matrix metalloproteases (i.e., MMP2)) [70,71,89,90,91]. Previously, we reported that collagen IV-conveyed signals are essential cues for liver metastasis in several tumor types including UM and identified mediators of collagen IV signaling as potential therapeutic targets in the management of hepatic metastases [70]. In addition, ECM1 promotes migration and invasion by inducing EMT [89], and MMP-2 is recognized as a crucial contributor to liver metastasis [71]

Our proteomic analysis unravels other markers that could be valuable as diagnostic and prognostic tools (i.e., Nidogen1; NID1). NID1 is a basement membrane glycoprotein that is involved in ECM cellular interactions, cell migration and invasion, promotes melanoma metastasis, and is correlated with poor clinical outcomes. In our study, high levels of NID1 were found in EVs derived from OMM2.5 (metastatic) cells. Previously, NID1 has been proposed as a new biomarker for disease progression and therapeutic target in breast cancer and melanoma [92].

In this study, we used an in vivo model to test whether UM-EVs could promote tumorigenesis. As shown by our group previously, exposure of cancer patient-derived EVs to single-oncogene mutated cells (such as Fibro-BKO, HEK 293 and PTEN KO MCF) resulted in malignant transformation of the recipient cells and induction of tumors in vivo. Here we injected NOD-SCID mice with Fibro-BKO cells exposed to UM cell-derived EVs and found a similar effect: Fibro-BKO cells exposed to UM cell-derived EVs developed tumors in vivo, while those exposed to NCM-derived EVs did not. Our in vivo study provides evidence that EVs derived from UM cancer cells have the potential to promote tumorigenesis in primed cells.

The selective enrichment of metastatic factors and signaling pathway components in UM-derived EVs will contribute to our overall understanding of the regulatory networks involved in the establishment of the tumor microenvironment. This information will be helpful in elucidating the pathophysiological functions of tumor-derived EVs, and aid in the development of UM diagnostics and therapeutics. In light of the data shown here, further studies to assess the downstream pathways that are altered in recipient cells are needed. Furthermore, understanding the role EVs play in mediating pro-tumor and in particular pro-metastasis processes in target organs, such as the liver are needed. Finally, validation of protein signatures, and potential biomarkers, are needed in EVs isolated from UM patient blood.

## 4. Material and Methods

### 4.1. Cell Culture Conditions

Human eyes (*n* = 3, Table S1) were obtained from Centre universitaire d’ophtalmologie (Centre Hospitalier Universitaire de Québec, Canada), following an informed consent from the donor’s next of kin. Eyes were used in accordance to a protocol approved by the IRB of the Research Institute (RI) of the McGill University Health Centre (MUHC) (IRB #2019-5314).

NCM cultures were established from donor eyes [49,73]. In brief, the cornea, lens, vitreous humor and iris were removed. A total of four incisions were made toward the optic nerve to obtain a petal-like structure (Figure S1). The choroid was detached from the sclera, transferred into a solution of 0.02% EDTA at 37 °C for 30 min, and incubated in a mixture of collagenases IA and IV in trypsin (0.5 mg/mL each) (Sigma-Aldrich, St. Louis, MO, USA) to remove retinal pigment epithelium (RPE) cells. The choroid was then incubated in dispase II (Boehringer Ingelheim, Ingelheim am Rhein, Germany) and diluted in Melanocyte Growth Medium M2 medium (Promocell, Heidelberg, Germany) for 18 h at 37 °C. The reaction was stopped in Complete Protease Inhibitor Cocktail (Roche Diagnostics, Mannheim, Germany). Digested tissue was shaken to obtain single cell suspensions. Cells were passed through a 40 μm cell strainer, pelleted at 100 g for 5 min, resuspended in M2 medium, and cultured on FNC (0407, Athena, Baltimore, MD, USA) coated T25 tissue culture flasks. Culture medium was changed every three days. After reaching 80% confluency, cells were cryopreserved in liquid nitrogen using cryo-SFM (PromoCell, Heidelberg, Germany) for further usage. In the case of contamination with RPE cells or fibroblasts, culture medium was supplemented with 100 μg/mL geneticin (Sigma-Aldrich) for 7 days prior to subcultivation.

MP41, MP46 and 92.1 were purchased from ATCC (American Type Culture Collection). MEL270, MEL285 and OMM2.5 were kindly gifted by Dr. Vanessa Morales (University of Tennessee), human BRCA1-deficient fibroblasts were from Dr. Goffredo Arena and Immortalized human hepatocytes were gifted by Dr. Peter Metrakos (McGill University).

UM cell lines (MP41, MP46, 92.1, MEL 270, MEL285 and OMM2.5) were maintained in Roswell Park Memorial Institute media (RPMI 1640) supplemented with 10% Fetus Bovine Serum, 0.1% 10 U/mL penicillin and 10 μg/mL streptomycin, 4 mM L-glutamine and 10 μg/mL insulin, 1 mM NaPyruvate. All the media components were purchased from Corning. Human BRCA1-deficient fibroblasts (Fibro-BKO) [47] and immortalized human hepatocytes (IHH) were maintained in DMEM-F12 medium supplemented with 10% FBS, and penicillin-streptomycin antibiotics.

### 4.2. EV Isolation

UM cell lines were cultured in T75 flasks until they reached 80% confluency; then cell culture medium was replaced by medium supplemented with EV-depleted FBS. NCM cells were cultured in T25 flasks in M2 medium until they reached 80% confluency; then medium was changed with fresh M2 medium. Cells were allowed an additional 24 h incubation before conditioned media collection. Conditioned media from all cell cultures were subjected to a series of sequential differential centrifugation steps. The supernatants were centrifuged at 500× *g* for 10 min to remove contaminating cells, followed by centrifugation at 2000× *g* for 20 min to remove cell debris. Supernatants were passed through a 0.2 μm syringe filter (Corning), transferred to 26.3 mL polycarbonate tubes (# 355618; Beckman Coulter), and centrifuged at 16,500× *g* for 20 min at 4 °C to remove apoptotic bodies and cell debris. Supernatants were transferred to new 26.3 mL polycarbonate tubes and ultracentrifuged at 120,000× *g* (40,000 rpm) for 70 min at 4 °C using 70 Ti rotor in Optima XE ultracentrifuge machine (Beckman Coulter). The crude EVs pellets were washed with PBS at 120,000× *g* for 70 min at 4 °C, resuspended in 500 μL PBS, and stored in −80 °C until use.

For proteomic analyses of EVs, samples were purified using iodixanol (OptiPrep^TM^ density gradient [93,94] (Sigma-Aldrich). Briefly, a density gradient was prepared by serial dilutions of iodixanol stock (60% *w*/*v*): (i) five volumes of 60% iodixanol were mixed with 1 volume of 0.25 M sucrose, 0.9 M NaCl and 120 mM HEPES solution (pH 7.4) to obtain 50% iodixanol, (ii) 2 volumes of 50% iodixanol were mixed with three volumes of 0.25 M sucrose, 150 mM NaCl and 20 mM HEPES (SNH) solution (pH 7.4) to obtain 20% iodixanol, and (iii) 1 volume of 50% iodixanol was mixed with 9 volumes of SNH solution to prepare 5% iodixanol. EVs in 1.92 mL of HEPES-buffer were mixed with 2.88 mL of 50% iodixanol to obtain EVs-containing 30% iodixanol solution. The discontinuous iodixanol density gradient was prepared by carefully layering 2.5 mL of 5% iodixanol and 3 mL of 20% of iodixanol sequentially with the help of stainless steel 316 syringe needle (Sigma-Aldrich) in 13.26 mL Ultra-Clear tubes (Beckman Coulter). EVs in 30% iodixanol solution (4.8 mL) were carefully placed at the bottom of the tubes containing the iodixanol gradients using the syringe needle without disturbing the gradient. Tubes were spun at 38,000 rpm for 2 h using SW41 Ti swinging bucket rotor in LS8 ultracentrifuge at 4 °C. One millilitre fractions were collected (*n* = 10), diluted in PBS and centrifuged at 120,000× *g* for 70 min. The pellet of each fraction was resuspended in PBS and stored for EV characterization (i.e., NanoSight for nanoparticle tracing analysis (NTA) (Figure S8), Western blot (Figure S9) and transmission electron microscopy (TEM) (Figure S10)). In parallel, a control iodixanol density gradient was centrifuged with the sample. After centrifugation, 1 mL fractions were collected and 100 μL of each fraction was added to 96-well plates to read the absorbance at 340 nm to determine its density. Fraction 3 was enriched in EVs and was used for further analyses (Figure S5)

### 4.3. EV Characterization: TEM and Size Distribution Analyses

For TEM, EVs were processed in 0.1% sodium cacodylate washing buffer (250 mL EMS, 35× *g* sucrose, 250 mL water) and centrifuged at 120,000× *g* for 70 min. EV pellets were resuspended in 2.5% glutaraldehyde fixation solution (250 mL EMS, 50 mL 25% glutaraldehyde, 250 mL water). 10 μL of fixed EVs was put on TEM copper grids and left to settle for 20 min. Grids were washed with 0.02 M glycine for 10 min, and EVs were blocked in 2% bovine serum albumin/2% casein/0.5% ovalbumin solution. Primary antibodies (CD63, CD81, TSG101) were applied for overnight incubation at 4 °C in blocking buffer in 1:1 ratio. Grids were washed in Dulbecco’s PBS (DPBS) and incubated with 20 nm gold anti-mouse (ab27242) and 10 nm gold anti-rabbit-conjugated secondary antibodies (ab272234) (1:20 ratio) at room temperature for 30 min. After washing with DPBS, EVs were stained with 4% uranyl acetate for 3 min and air-dried overnight. The grids are examined using FEI Tecnai^TM^ G2 Spirit BioTwin 120 kV Cryo-TEM. To quantitatively assess the size of the EVs, at least 100 vesicles were counted. In parallel, an aliquot of 5 μL of EVs sample was run on a Nanosight NS500 system (Nanosight Ltd., Amesbury, UK), and the concentration and size distribution was analyzed using the NTA 1.3 software (Malvern Panalytical).

### 4.4. Cell Exposure to EVs

Fibro-BKO were used as target cells to analyze the biological effects of EVs isolated from NCMs and UM cell lines. When Fibro-BKO reached 30% confluence, they were treated with complete DMEM-F12 medium supplemented with 10^8^ EVs/mL. At 80–90% cell confluency, cells were passaged 1 in 6 using 0.05% Trypsin-EDTA (Wisent). Cell count and viability was assessed using a hemocytometer and trypan blue exclusion staining.

### 4.5. Population Doubling Level (PDL) Calculation

Cells were considered at population doubling zero at the first time they were exposed to NCM-EVs or UM-EVs. At every passage, cell number was determined and population doubling was calculated using the following formula; PDL  =  log(Nh/Ni)/log2, where Nh is the number of cells harvested at the end of the incubation time and Ni is the number of cells inoculated at the beginning of the incubation time. Cumulative PDL was calculated by adding the previous calculated PDL.

### 4.6. EV Labeling and Cellular Uptake Assay

Isolated EVs were labeled with PKH67 green fluorescent probe according to the manufacturer’s instructions (Sigma-Aldrich). Briefly, EVs were resuspended in Diluent C and mixed with equal volume of the stain solution (4 μL PKH 67 in 1 mL Diluent C) for 5 min. The reaction was stopped by adding 2 mL of 2% BSA. Control samples, consisting of EVs-free medium with Diluent C were run in parallel. All samples were passed through OptiPrep^TM^ density gradient to purify EVs from unbound PKH67 dye. Fraction 3 (enriched in EVs, see previous sections) was collected from both control (PBS + PKH67 dye) and samples (EVs + PKH67 dye), and centrifuged at 120,000× *g* for 70 min at 4 °C. Pellets were resuspended in culture medium and added to Fibro-BKO and IHH cultures in four-well chamber slides (Ibidi, Gräfelfing, Germany) for 6 h. Stained cells were visualized using an LSM780 confocal microscope (Carl Zeiss, Oberkochen, Germany).

### 4.7. Immunofluorescence

Cells (20,000) were plated in four-well chamber slides for 24 h, fixed with 4% paraformaldehyde for 30 min, permeabilized with 0.1% Triton X for 15 min, and incubated in blocking buffer (1% BSA in PBS) for 1 h. Primary antibodies against MLANA (MelanA) (TA801623), Vimentin (ab92547), HMB45 (sc-59305), S100 (ab4066), cytokeratin 18 (ab32118), cytokeratin 8 (ab59400) were added to cells at 1:1000 in blocking buffer and incubated overnight at 4°C. Slides were washed 5 times in PBS, and cells were counterstained for 1 h with fluorophore-conjugated secondary antibodies (1:1000 in blocking buffer). Cells were washed in PBS and the slides were mounted on coverslips with NucBlue (R37605; ThermoFisher, Waltham, MA, USA). Cells were visualized using an LSM780 confocal microscope.

### 4.8. Migration Assay

Cell migration was evaluated using wound-healing assay. Briefly, Fibroblast-BKO cells were seeded into 12 well plate at a density of 0.5 × 10^6^ cells per well. After reaching confluence, the monolayers were scratched using 200 μL pipette tips and washed twice with PBS. Fresh complete media was added with EVs (NCM-EVs, MEL270 UM-EVs, OMM2.5 UM-EVs) or without EVs (PBS, vehicle) and photographed at 0 h and 12 h using inverted microscope at 100× magnification. The areas of wound closure were calculated using ImageJ software (ImageJ 1.53a, https://imagej.nih.gov/ij/). The wound-healing assay was carried out in triplicate (*n* = 3).

### 4.9. Transwell Invasion Assay

Invasion assay was conducted using the croning^®^ BioCoatTM Matrigel^®^ invasion chamber (354480) according to the manufacturer’s protocols. Briefly, the inserts were rehydrated with DMEM medium for 2 h in the incubator. A total of 5 × 10^5^ Fibroblast BKO cells pretreated with EVs derived from NCM (2G.PPccF68Y), MEL270 and OMM2.5 or control (without EVs treatments), were seeded onto the matrigel coated insert in 400 μL serum-free DMEM. Then, 750 μL of DMEM with 10% FBS was added into the lower well of the Transwell chamber. After 24hrs of incubation in the incubator (at 37 °C, 5% CO_2_), the membrane of the insert was stained with a staining solution (Millipore Sigma, ECM508) for 20 min at room temperature. The inserts were washed twice with distilled water and the cells on the upper surface (non-invaded) of the insert were removed by scrubbing with cotton tipped swabs and washed away with distilled water. Finally, the invaded cells were counted under the inverted microscope at 100X magnification. At least five images were taken at random location per insert and transwell invasion assay was performed in triplicates (*n* = 3).

### 4.10. Protein Isolation from Cells and EVs

EVs and cells were lysed using RIPA buffer supplemented with Complete^TM^ mini protease inhibitor (Sigma-Aldrich) at 4 °C for 30 min. Samples were pulse sonicated for 2 s (three times), and were spun at 13,000× *g* for 30 min at 4 °C. Supernatants were quantified using micro BCA and Pierce BCA (Fisher Scientific) for EVs and cells, respectively. Proteins samples were processed for Western blot and proteomic analyses by mass spectrometry.

### 4.11. Western Blot

25 μg of cell proteins and 10 μg of EVs proteins were separated using 12% precast polyacrylamide gel and transferred onto polyvinylidene fluoride (PVDF) membranes (BioRad, Hercules, CA, USA). Membranes were blocked for 1 h in 5% non-fat dry milk in Tris buffer saline with 0.05% Tween-20 (TBST). Membranes were probed with anti-TSG101 (1:1000) (ab125011), anti-CD63 (1:1000) (ab59479), anti-CD81 (1:1000) (ab109201) GM130 (ab59400) (all from abcam, Cambridge, UK), and β-actin (1:10000) (A2228-200 μL) (Sigma-Aldrich). Membranes were washed in TBST and were treated with corresponding horseradish peroxidase-conjugated secondary antibodies anti-rabbit hP (7074S) and anti-mouse hP (7076P2) (Cell Signalling Technology, Denver, MA, USA). Blots were developed using ECL prime Western blot detection (GE healthcare, Chicago, IL, USA) and visualized using the ChemiDoc^TM^ XRS+ System (Biorad, Hercules, CA, USA).

### 4.12. Label-Free LC-MS/MS Proteomics Analysis of EVs and Database Search

LC-MS/MS proteomic analyses were done on EVs proteins (10 μg) as previously described [95]. Samples were run in triplicates. Raw data were converted into *.mgf (Mascot generic format) to use the Mascot2.6.2 search engine (Matrix Science, London, United Kingdom) to search against human protein sequences (Uniprot 2019). The database search results were loaded onto Scaffold Q + Scaffold_4.10.0 (Proteome Sciences, London, United Kingdom) for spectral counting, statistical treatment, data visualization and quantification. Additional filters were applied, such as protein identification which was considered if they had quantifiable protein area in 2 or more of the biological triplicates, protein threshold greater than 99.0%, and peptide threshold greater than 95.0%. Samples with low total protein counts and low spectrum counts were excluded from the analyses. The identified protein list in Scaffold were exported to Microsoft Excel and uploaded into the DAVID Bioinformatics database (Database for Annotation, Visualization and Integrated Discovery version 6.8) [95,96] for the analysis of functional gene enrichment and annotation, and KEGG pathway. In addition, bioinformatic analysis and Vesiclepedia database search were performed using the FunRich software (Functional Enrichment Analysis Tool version 3.1.3) [97].

### 4.13. In Vivo Tumor Growth

The 5-week-old female NOD-SCID mice (Jackson Laboratory) were used with approval and in compliance with the MUHC Animal Compliance Office (Protocols 2012–7280). All animal protocols were carried out according to institutional guidelines. Cells growing in log phase were harvested by trypsinization and washed twice with HBSS. Mice (*n* = 2 per group) were injected subcutaneously in the flanks with 2 million cells in 200 μL HBSS/Matrigel mixture. Mice were followed for tumor growth, and sizes of the xenotransplants were determined at euthanasia.

### 4.14. Immunohistochemistry Labelling Procedure

Mice xenotransplants were collected, fixed in 10% buffered formalin, embedded in paraffin, and stained with H&E (hematoxylin and eosin) according to standard protocols or processed for immunohistochemistry. Briefly, 5 μm tissue sections were dewaxed in xylene and rehydrated with distilled water. After antigen unmasking and blocking of endogenous peroxidase (3% hydrogen peroxide), the slides were incubated with rabbit anti-Ki67 (abcam, ab15580), rabbit anti-MelanA (abcam, ab51061) or rabbit anti-Vimentin (abcam, ab92547) primary antibodies. Labeling was performed using EnVision+ System-HRP anti-rabbit (Dako, K4003) and the Liquid DAB+ Substrate Chromogen System (Dako, K3468). Sections were counterstained lightly with Hematoxylin before mounting.

### 4.15. Statistical Analysis

Data were analyzed using Student’s *t* test for unpaired samples. The criterion for significance (*p* value < 0.05) was set as mentioned in figures.

## 5. Conclusions

Metastasis is rarely found during diagnosis of primary UM and many patients already have organ specific micrometastases by the time the ocular tumor is detected [98]. Moreover, CTCs have been detected at primary UM diagnosis, preceding the clinical detection of metastasis [99]. There remains an urgent need for tools that will aid in the screening and monitoring of tumor burden. As the molecular contents of EVs reflect their cellular origin, EVs derived from cancer patient plasma can prove vital to the understanding of tumor progression, metastatic risk and allow real time evaluations of therapeutic outcome. This ability renders them prone to be used in liquid biopsy for detection of cancer biomarkers [100,101]. In the present study, we profiled the proteome of pure preparations of EVs in the context of UM and characterized their behaviour. The next step is to perform a comparative proteome profiling of EVs derived from both healthy individuals and from patients presenting with either uveal nevi or melanoma, with the ultimate goal of developing a non-invasive method to detect UM metastasis with high sensitivity and specificity.

## Supplementary Materials

The following are available online at https://www.mdpi.com/2072-6694/12/10/2923/s1 Figure S1: Isolation and maintenance of normal choroidal melanocytes (NCM). Figure S2: Phenotypical characterization of cultured human NCM. Figure S3: Nanoparticle tracking analysis of EVs derived from UM and NMC cells. Figure S4: Transmission electron microscope images displayed represented data of EVs derived from UM and NCM cells. Figure S5: Purification of PKH67-labelled EVs. Figure S6: UM-EVs did not affect the viability of Fibro-BKO cells. Figure S7: Complete Western-blot PVDF membrane. Figure S8: Measuring size and concentration of EVs derived from NCM (blue), MEL270 UM-EVs (red) and OMM2.5 UM-EVs (black). Figure S9: Western blot analysis of common EV marker CD63 (A) and control beta actin (B). Figure S10: Immunoelectron microscopy images of EVs isolated from MP41 (A) and MEL270 (B) cell lines. Table S1: Cells used in this study. Table S2: Differentially Expressed Proteins between NCM-EVs and primary UM-EVs (*p* ˂ 0.05). Table S3: Differentially Expressed Proteins between primary MEL270 UM-EVs and metastatic OMM2.5 UM-EVs (*p* ˂ 0.05). Table S4A: DAVID analysis of upregulated proteins between NCM-EVs and primary UM-EVs. Table S4B: DAVID analysis of downregulated proteins between NCM-EVs and primary UM-EVs. Table S5A: DAVID analysis of upregulated proteins between primary MEL270 UM-EVs and metastatic OMM2.5 UM-EVs. Table S5B: DAVID analysis of downregulated proteins between primary MEL270 UM-EVs and metastatic OMM2.5 UM-EVs. Table S5C: DAVID analysis of upregulated proteins between primary MEL270 UM-EVs and metastatic OMM2.5 UM-EVs. Lists of proteins clustered into the DAVID subcategories referred to in Figure 8. Table S5D: DAVID analysis of downregulated proteins between primary MEL270 UM-EVs and metastatic OMM2.5 UM-EVs. Lists of proteins clustered into the DAVID subcategories referred to in Figure 8.
