# Supplementary material for: Uveal Melanoma-Derived Extracellular Vesicles Display Transforming Potential and Carry Protein Cargo Involved in Metastatic Niche Preparation
Source: Cancers (Basel). 2020 Oct 11;12(10):2923. doi: 10.3390/cancers12102923 (PMC7600758; doi:10.3390/cancers12102923)
Supplement: Supplementary file 1 [file cancers-12-02923-s001.pdf]

Supplementary Figures: Uveal melanoma-derived extracellular vesicles displayed transforming potential and carried protein cargo involved in metastatic niche preparation

Thupten Tsering, Alexander Laskaris, Mohamed Abdouh, Prisca Bustamante, Sabrina Parent, Eva Jin, Sarah Tadhg Ferrier, Goffredo Arena, Julia V. Burnier

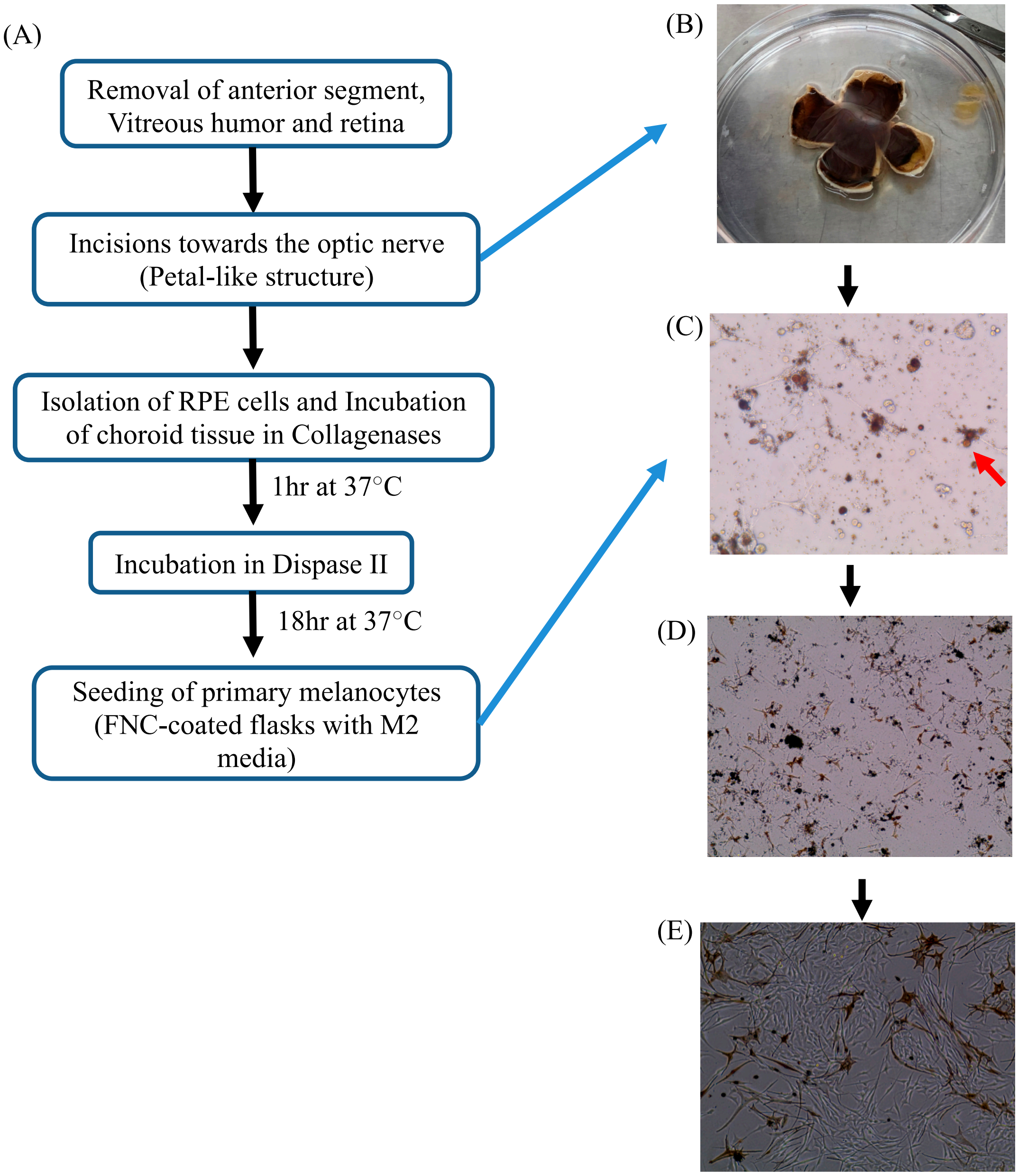

Supplementary Figure 1. Isolation and maintenance of normal choroidal melanocytes (NCM). **(A)** Summary of the procedure for NCM culture. **(B)** The eye was opened at the limbus using a scalpel and an incision was made to around the cornea. The anterior segment, vitreous and retina were removed, and four incisions were made to flatten the eyeball in a flower petal-like shape. **(C)** The choroid was detached from the sclera and incubated in a cocktail of collagenases IA and IV, followed by treatment with dispase II. **(D)** Cell suspension containing choroidal melanocytes was plated on FNC-coated flask containing serum free medium. Days after plating, clumps of RPE cells start growing. **(E)** Growing cells displayed spindle morphology and the presence of cytoplasmic pigmentation.

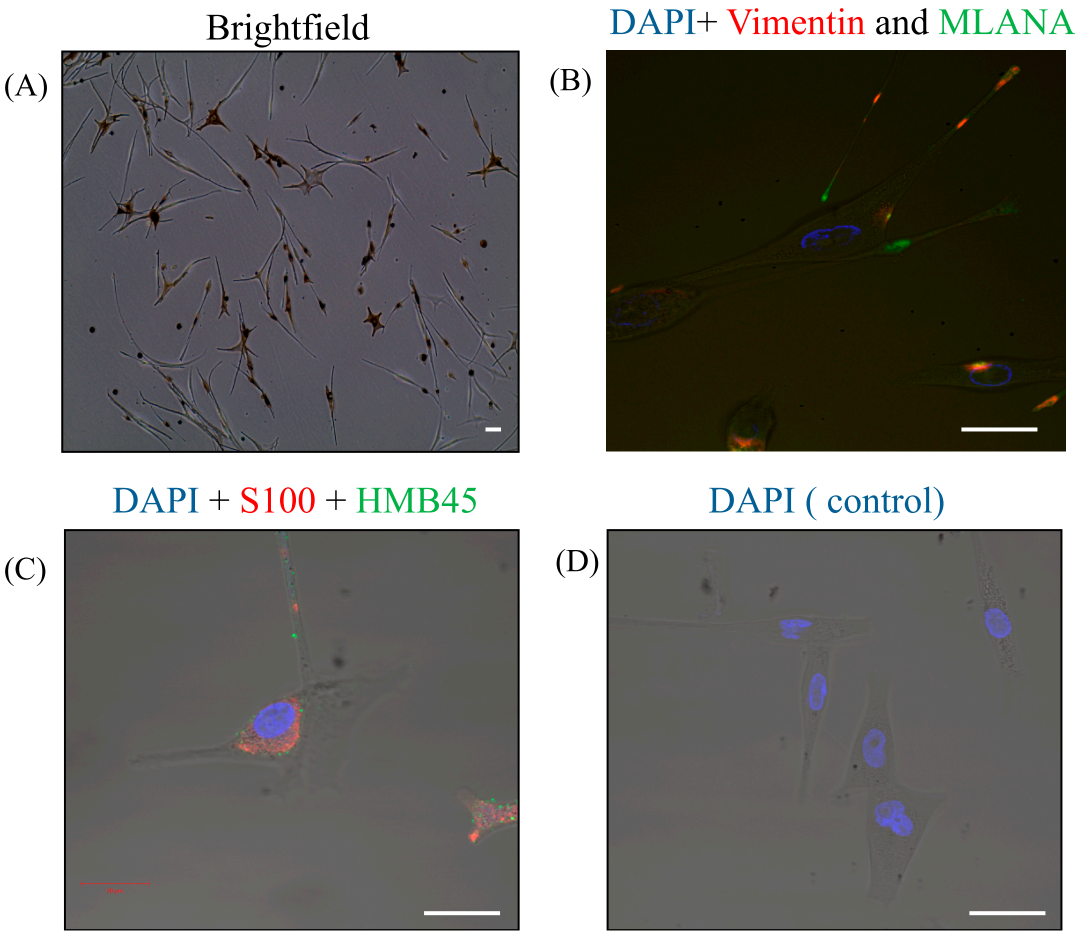

Supplementary Figure 2. Phenotypical characterization of cultured human NCM. **(A)** NCM observed under phase contrast microscopy displayed spindle shape and cytoplasmic pigmentation. **(B)** NCM labeled with anti-MLANA (green), Vimentin (red) and DAPI (blue; nuclei). **(C)** NCM labeled with S100 (red), HMB45 (green) and DAPI (blue). **(D)** NCM labeled with control isotypic primary antibodies. Presence of cells was revealed by DAPI staining. Scale bars: 20 µm.

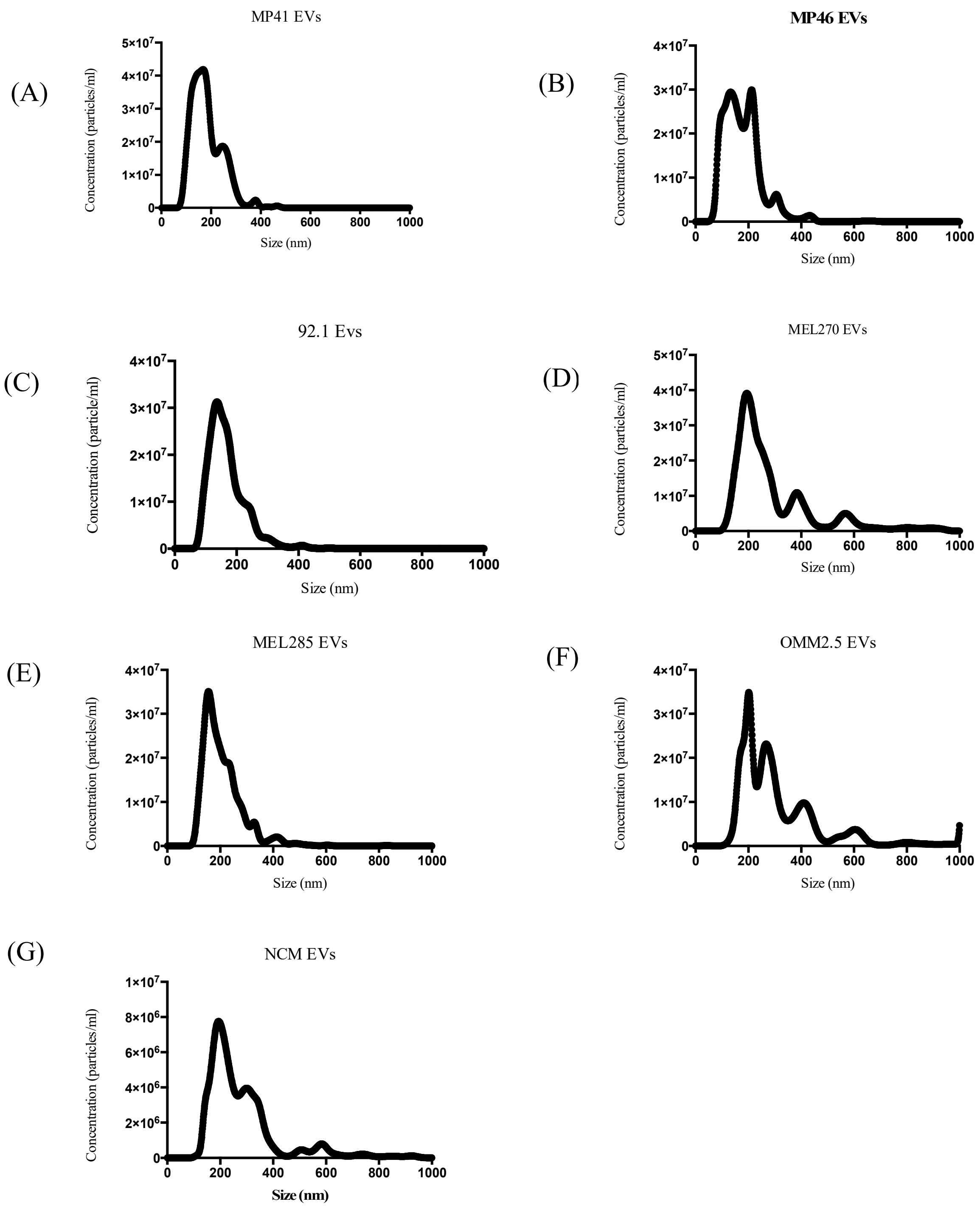

Supplementary Figure 3. Nanoparticle tracking analysis of EVs derived from UM and NMC cells. **(A)** MP41 EVs. **(B)** MP46 EVs. **(C)** 92.1 EVs. **(D)** MEL270 EVs. **(E)** MEL285 EVs. **(F)** OMM2.5 EVs. **(G)** NCM EVs.

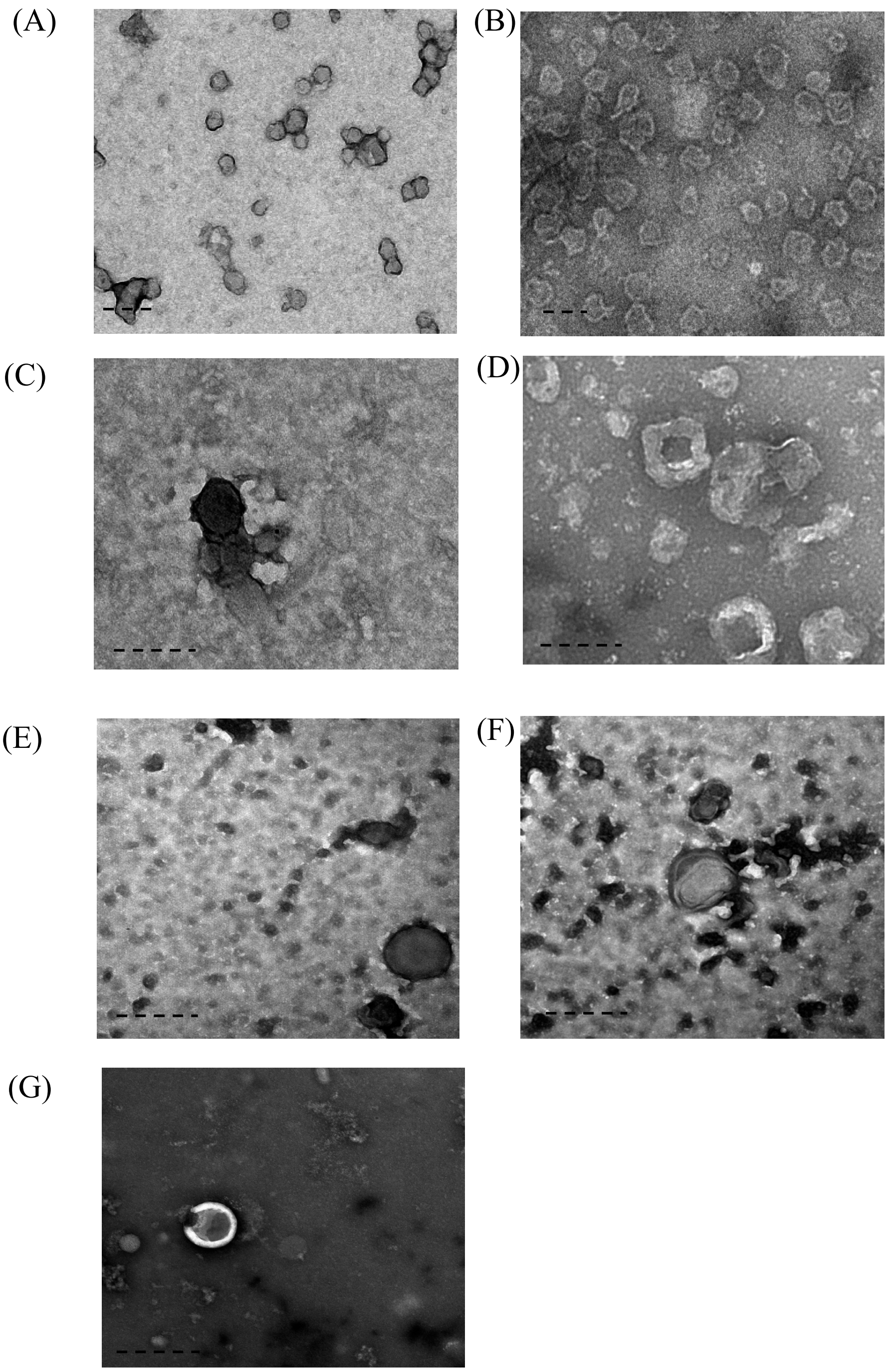

Supplementary Figure 4. Transmission electron microscope images displayed represented data of EVs derived from UM and NCM cells. **(A)** MP41 EVs. **(B)** MP46 EVs. **(C)** 92.1 EVs. **(D)** MEL270 EVs. **(E)** MEL285 EVs. **(F)** OMM2.5 EVs. **(G)** NCM EVs. Scale bars: 200 nm.

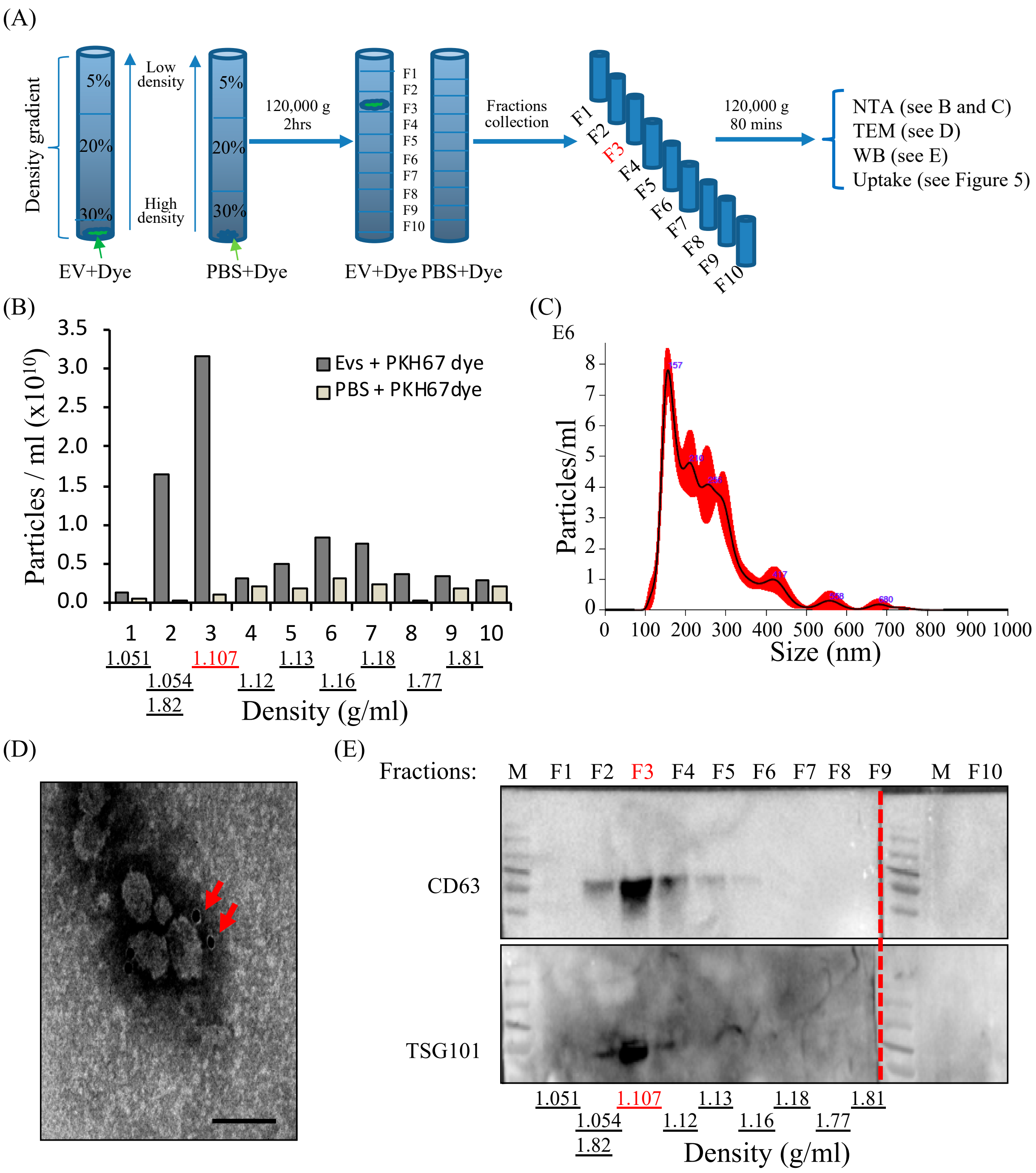

Supplementary Figure 5. Purification of PKH67-labelled EVs. **(A)** Workflow for the purification of EVs from excess PKH67 dyes using Optiprep density gradient and ultracentrifugation. **(B)** NTA analysis confirmed that EVs settled at the gradient fraction 3 (1.107 g/ml). **(C)** Purified EVs from fraction 3 displayed a mean size of 157 nm. **(D)** Representative micrographs of immunoGold-TEM on purified EVs from fraction 3 that were labelled with antibodies against CD63 (red arrows). Scale bars 200 nm. **(E)** Proteins isolated from different gradient fractions were analyzed by Western blot for the expression of specific exosome markers. The highest expression levels of exosome markers are located in fraction 3. M = Marker.

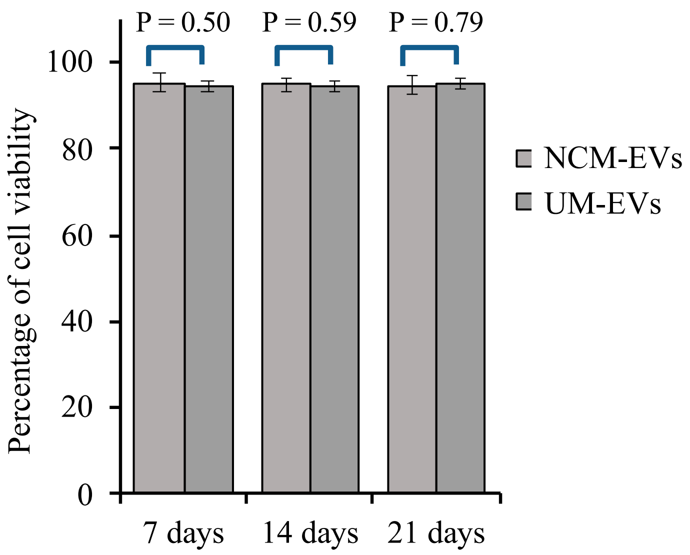

Supplementary Figure 6. UM-EVs did not affect the viability of Fibro-BKO cells. Fibro-BKO cells were cultured for 3 weeks in the presence of NCM-EVs or UM-EVs. Cells were analyzed for their viability by counting the number of viable cells over all counted cells using trypan blue staining. No significant difference was observed. Data are mean ± SD. P values are represented on the column graphs.

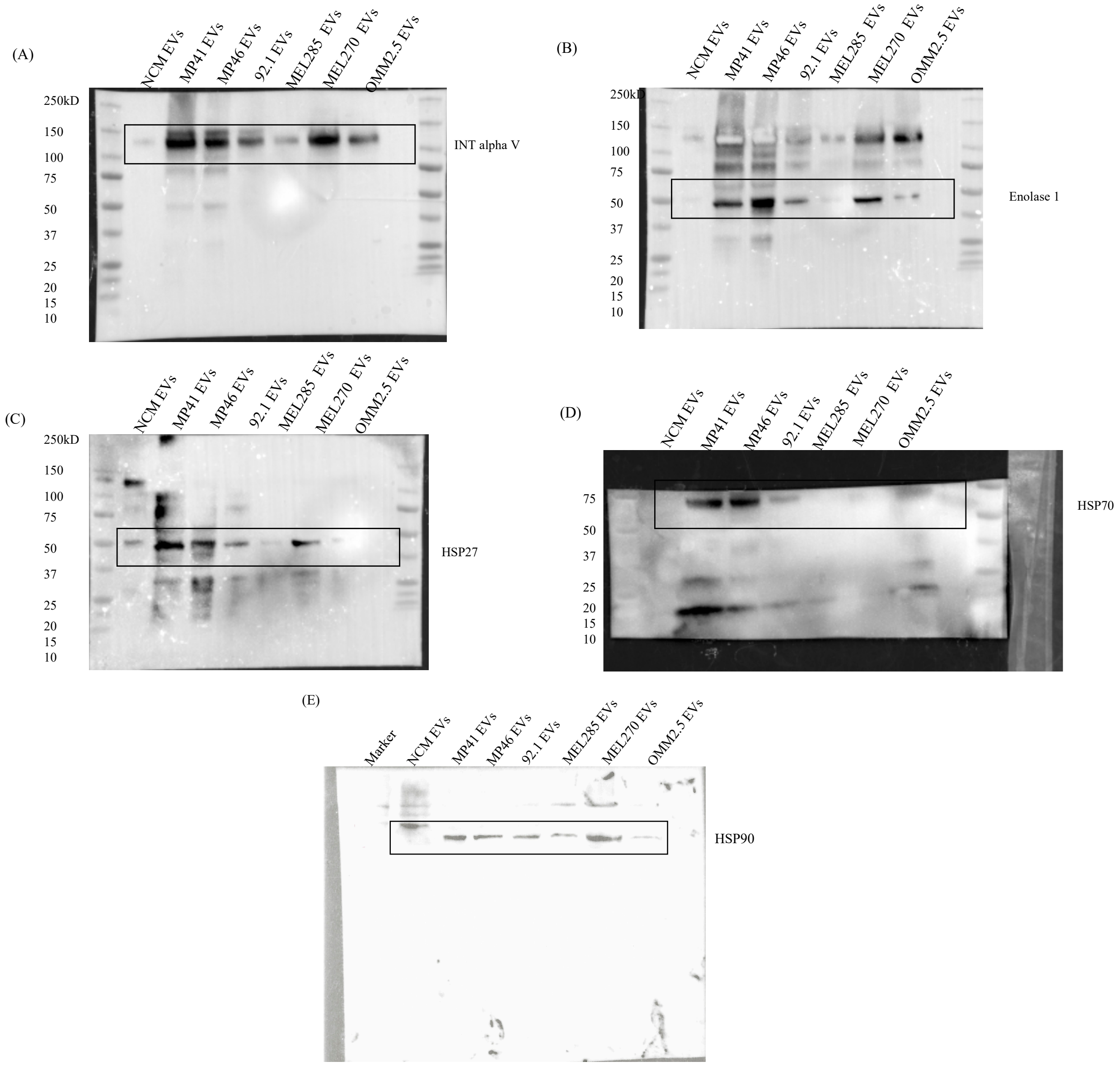

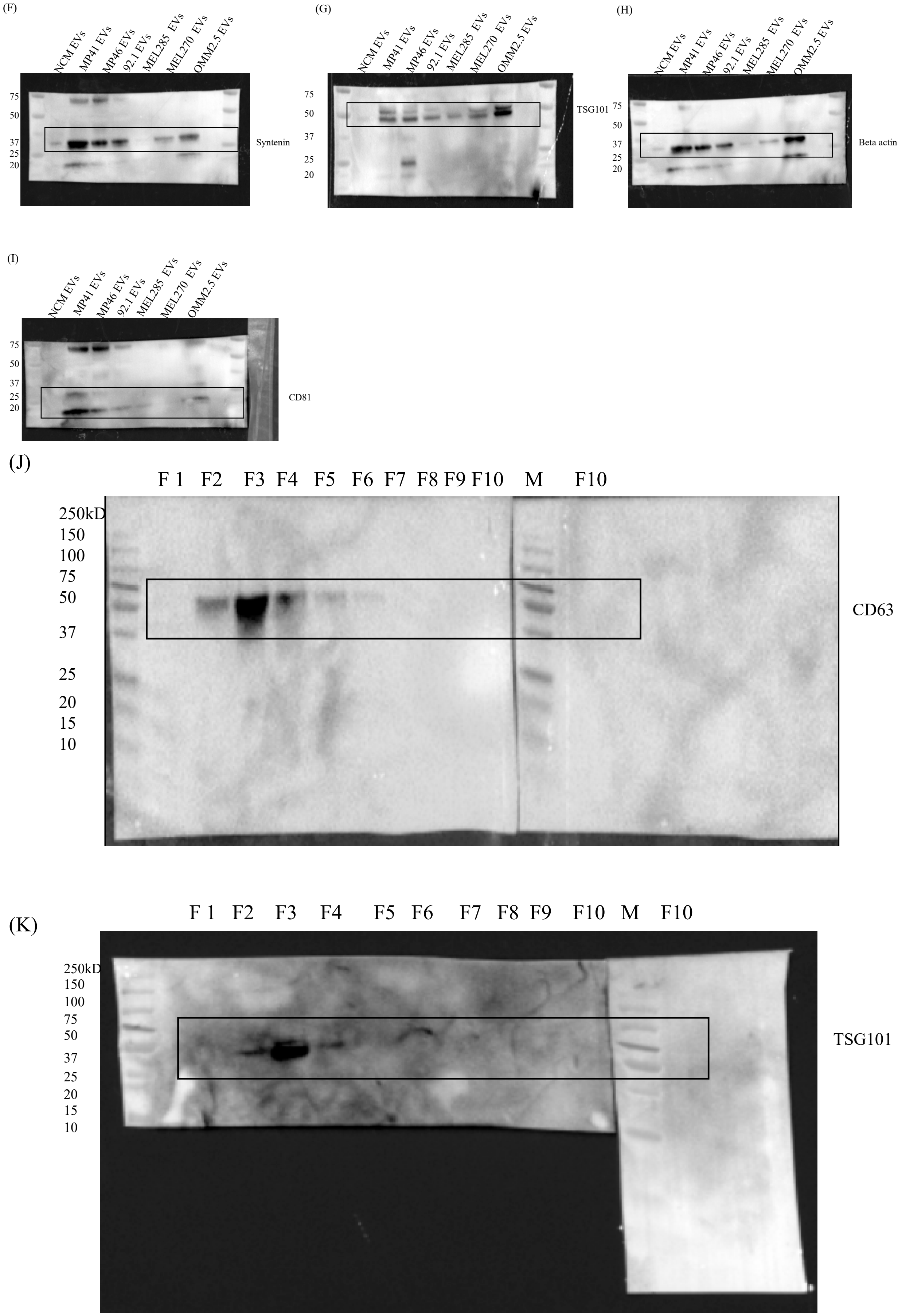

Supplementary Figure 7. Complete western-blot PVDF membrane **(A-K)**. **NOTE: (E)** HSP90 antibody was probe on a separate membrane. **(F)(G)(H)(I)** the membrane was cut prior to incubation of primary antibody in order to save antibodies.

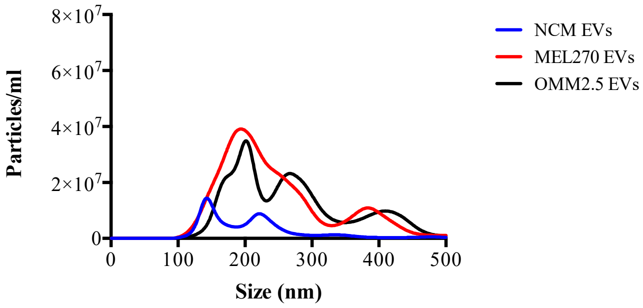

Supplementary Figure 8. Measuring size and concentration of EVs derived from NCM (blue), MEL270 EVs (red) and OMM2.5 EVs (black). x-axis range 0-500nm.

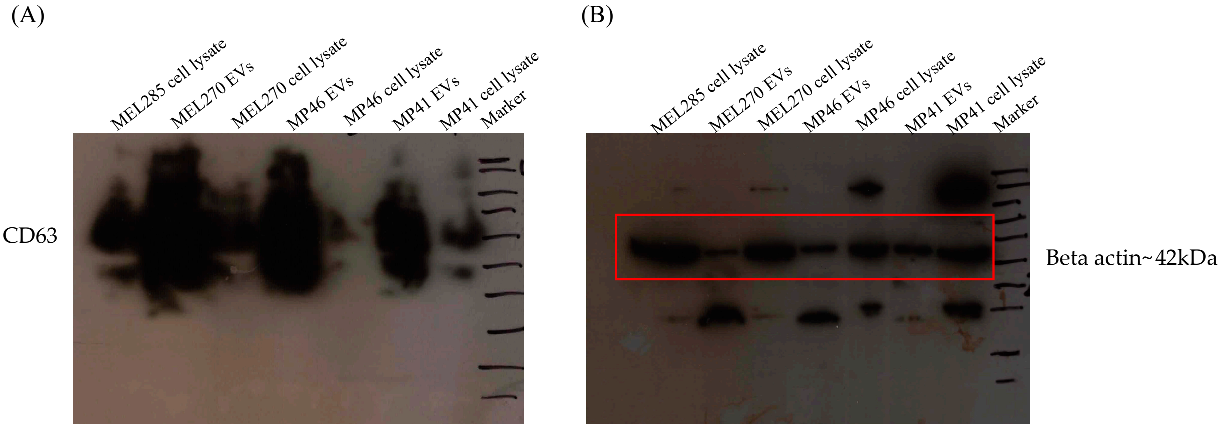

Supplementary Figure 9. Western blot analysis of common EV marker CD63 (A) and control beta actin (B).UM cell lines (MP41, MP46 and MEL270 cell lysates) and UM-EVs proteins were loaded into the 12% precast polyacrylamide gel. UM-EVs were enriched in CD63 marker compared to cell lysate.

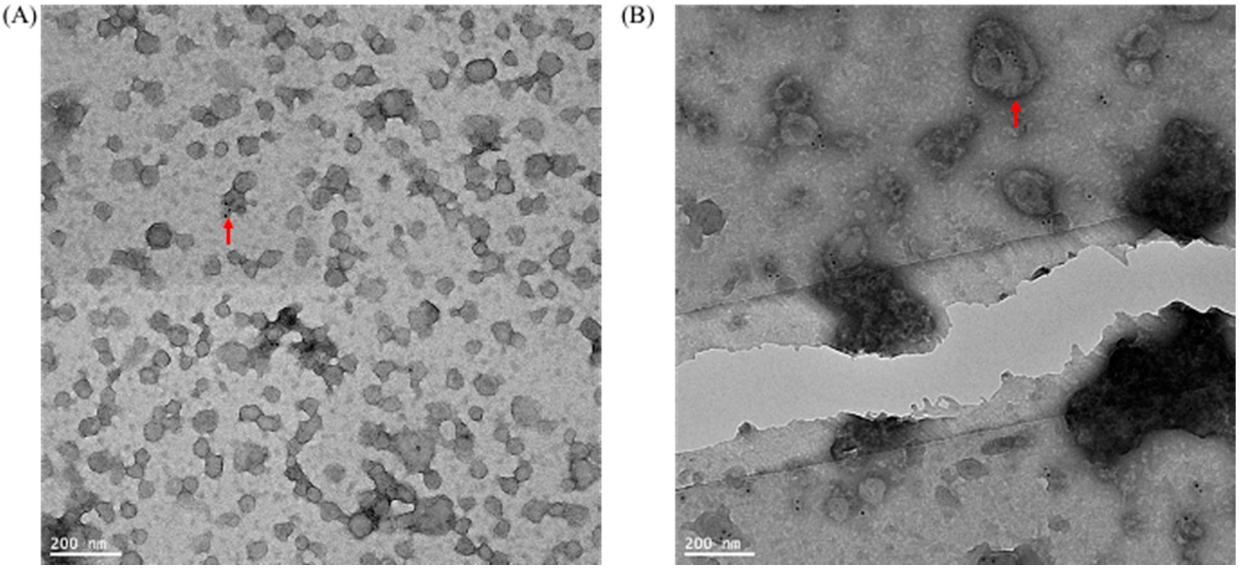

Supplementary Figure 10. Immunoelectron microscopy images of EVs isolated from MP41 (A) and MEL270 (B) cell lines. Red arrow indicating the 10nm gold labelled EVs with anti-CD81 antibody. (Magnification=29000 X, Scale bars - 200nm). Note, MEL270 UMEVs are bigger compared to MP41 UM-EVs.
